# Anchoring cortical granules in the cortex ensures trafficking to the plasma membrane for post-fertilization exocytosis

**DOI:** 10.1038/s41467-019-10171-7

**Published:** 2019-05-22

**Authors:** Edgar-John Vogt, Keizo Tokuhiro, Min Guo, Ryan Dale, Guanghui Yang, Seung-Wook Shin, Maria Jimenez Movilla, Hari Shroff, Jurrien Dean

**Affiliations:** 1 Laboratory of Cellular and Developmental Biology, NIDDK, National Institutes of Health, Bethesda, MD 20892 USA; 2 Section on High Resolution Optical Imaging, NIBIB, National Institutes of Health, Bethesda, MD 20892 USA; 3 Department of Cell Biology and Histology, Medical School, University of Murcia, IMIB, 30100 Murcia, Spain; 4 Advanced Imaging and Microscopy Resource, National Institutes of Health, Bethesda, MD 20892 USA; 5 Present Address: Charité - Universitätsmedizin Berlin, Augustenburger Platz 1, 13353 Berlin, Germany; 6 Present Address: Department of Genome Editing, Institute of Biomedical Science, Kansai Medical University, 2-5-1 Shinmachi, Hirakata, Osaka 573-1010 Japan; 7 Present Address: Bioinformatics and Scientific Programming Core, Eunice Kennedy Shriver National Institute of Child Health and Human Development, National Institutes of Health, Bethesda, MD 20892 USA

**Keywords:** Super-resolution microscopy, Actin, Exocytosis

## Abstract

Following fertilization, cortical granules exocytose ovastacin, a metalloendopeptidase that cleaves ZP2 in the zona pellucida surrounding mouse eggs to prevent additional sperm binding. Using high- and super-resolution imaging with ovastacin^mCherry^ as a fluorescent marker, we characterize cortical granule dynamics at single granule resolution in transgenic mouse eggs. Newly-developed imaging protocols provide an unprecedented view of vesicular dynamics near the plasma membrane in mouse eggs. We discover that cortical granule anchoring in the cortex is dependent on maternal MATER and document that myosin IIA is required for biphasic trafficking to the plasma membrane. We observe local clearance of cortical actin during exocytosis and determine that pharmacologic or genetic disruption of trafficking to the plasma membrane impairs secretion of cortical granules and results in polyspermy. Thus, the regulation of cortical granule dynamics at the cortex-plasma membrane interface is critical for exocytosis and the post-fertilization block to sperm binding that ensures monospermic fertilization.

## Abstract (web summary)

Mammalian eggs require a single sperm for viable fertilization, and cortical granule exocytosis prevents additional sperm binding. Vogt et al. image at single granule resolution to document that cortical granule anchoring in the cortex ensures proper trafficking, exocytosis and polyspermy block.

## Introduction

Regulated exocytosis is a fundamental cellular event that releases cargo molecules at the cell surface for specific physiological tasks including neurotransmission, immune response, and reproduction^1^. In mouse eggs, exocytosis of cortical granules (CGs) makes the extracellular zona pellucida impermeable to additional sperm in the post-fertilization block to polyspermy^2^. CGs are derived from the Golgi apparatus and migrate towards the cortex during oogenesis where they accumulate in a uniform layer that is displaced 0.4 to 0.6 μm inward from the plasma membrane^3^. Anchoring CGs at the periphery is correlated with the acquisition of exocytotic competence, defined as the ability to undergo exocytosis in response to increased intracellular calcium^4^. In mice, the transport of CGs to the cortex is dependent on actin^5^ and the egg cytoplasmic actin network mediates long-range transport of vesicles^6^. A recent study carefully documented two pathways that translocate CGs from the center of the egg to the periphery during meiosis. One depends on myosin Va powered movement along actin filaments and the other relies on CG binding to Rab11a vesicles to hitchhike to the egg cortex. Defects in these pathways lead to polyspermy^7^.

However, our current understanding of CG dynamics at the cortex-plasma membrane interface in mouse eggs remains limited. In the absence of protocols to perform live-imaging near the plasma membrane, earlier studies relied on staining fixed CGs with the relatively non-specific LCA (*lens culinaris* agglutinin) lectin. In additional, mouse CGs are ~200 nm which is near the diffraction limit of standard light microcopy^8^. However, total internal fluorescence microscopy (TIRFM) combined with super-resolution structured illumination microscopy (SIM) can overcome these limitations and provide sub-diffractive, live-imaging of fluorescently labeled subcellular organelles^9–11^.

We have previously reported a subcortical maternal complex (SCMC) that forms during oogenesis and, when disrupted, delays or abrogates cleavage-stage embryogenesis^12–16^. Its biological function(s) during oogenesis have been less well investigated and its peripheral location suggests potential involvement in membrane-associated processes including vesicle trafficking and exocytosis. We also have reported on ovastacin, a zinc metalloendopeptidase that was identified as a pioneer marker of mammalian CGs. It is released during exocytosis and cleaves ZP2 in the extracellular zona pellucida to prevent sperm binding which provides a potent post-fertilization block to polyspermy^17,18^.

Here, we seek to gain insight into CG biology using multiple live-cell imaging modalities with enhanced spatiotemporal resolution and transgenic mice expressing fluorescently tagged ovastacin^mCherry^ as a marker of CGs. We genetically document the involvement of the SCMC component MATER in anchoring CGs at the egg cortex and demonstrate a role for myosin IIA in CG trafficking and clearance of cortical actin prior to exocytosis. Perturbation of CG trafficking at the cortex-plasma membrane interface leads to polyspermy and adversely affects in vivo female fertility.

## Results

### Mouse CGs accumulate in the egg cortex

To perform live-diffraction limited and super-resolution (instant TIRF-SIM) imaging of the cortical cytoskeleton and plasma membrane, the 8 μm thick zona pellucida was removed (Supplementary Fig. 1a–d). Live confocal microscopy of stimulated wild-type eggs in the presence of plasma membrane (CellMask) and actin-binding fluorogenic (SiR-actin) dyes documented an uneven plasma membrane topography and enrichment of filamentous actin (F-actin, referred to as actin hereafter) as a cytoskeletal scaffold. Single XZ optical sections spanning the membrane clusters showed individual microvilli-like structures and actin fibers that extended in the axial direction. 3D recordings further documented dynamic disappearance and reappearance of actin in a single lateral plane (Supplementary Fig. 2a–f).

**Fig. 1:**
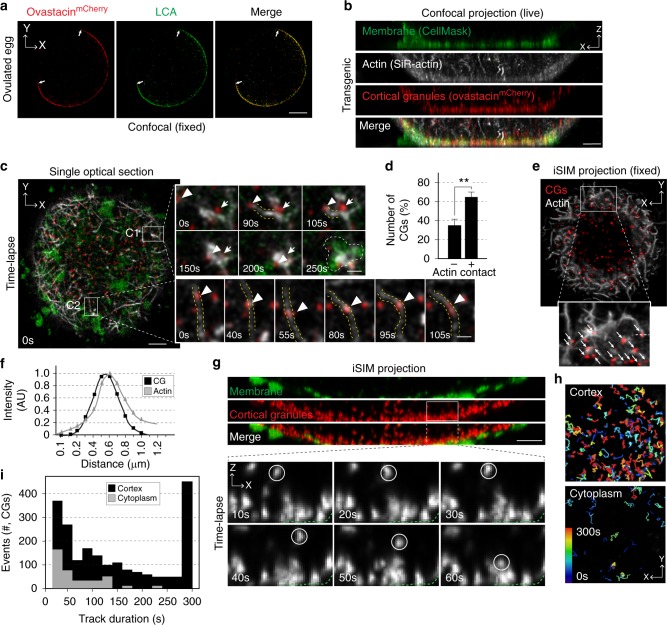
Cortical granules in the mouse egg. **a** Confocal cross-sections of z-projections of individual and merged channels of ovastacin^mCherry^ eggs (red) fixed and stained with LCA-FITC (green). Arrows, cortical granule free zone. Scale bar, 20 μm. **b** XZ projection of XY optical sections (0.2 μm) of zona-free ovastacin^mCherry^ eggs stimulated to exocytose cortical granules (red). Microvillar plasma membrane (green), actin (gray). Scale bar, 5 μm. **c** A XY optical section of transgenic eggs sampled every 5 s (×50). Enlarged views (right) at indicated time points. C1, a stationary cortical granule (red, arrow) and a cortical granule (red, arrowhead) captured and pulled by retracting F-actin (gray, dashed yellow line). Plasma membrane (green) accumulates in a reservoir (dashed white line). C2, a cortical granule (red, arrowhead) moving along an actin structure (gray, dashed yellow line) Scale bars, 5 μm (left), 2 μm (right). See corresponding Supplementary Movies 1,2. **d** Quantification of cortical granules in (+; *n* = 415) and not in (-; *n* = 734) contact with actin. **p < 0.01 by two-tailed Student’s *t*-test. Error bar = s.e.m. The experiment reflects 3 biologically independent replicates. **e** Zona-free ovastacin^mCherry^ eggs were fixed 15 min after stimulation with SrCl_2_ and immunostained with phalloidin. iSIM projection of egg cortex with a magnified view (inset) showing single cortical granules (red, white arrows) juxtaposed with actin structures (gray). **f** Profile of dashed line in **e** (inset) indicating peaks in mCherry (CG, cortical granule) and phalloidin (actin) channels. **g** XZ projection spanning 8 μm. Volumes were acquired every 10 s at 0.2 μm steps for 5 min with iSIM. A representative iSIM image of an XZ projection of an egg with cortical granules (red) and the plasma membrane (green). Enlarged views at indicated time points corresponds to the white inset in the ovastacin^mCherry^ channel. A cortical granule (circle) approaches the plasma membrane (dashed green line). Scale bar, 5 μm. See corresponding Supplementary Movie 3. **h** First frame from iSIM time-lapse series in **g** with overlaid vesicle tracks in the cytoplasm (bottom) and cortex (top). Lines are colored to indicate time (lower left). **i** Histogram comparing distribution of cortical granules in the cytoplasm (*n* = 311) and cortex (*n* = 3710) during 5-min live-imaging period. Source data are provided as a Source Data file

CGs initially were detected by confocal microscopy in fixed eggs from transgenic mice expressing ovastacin^mCherry^ (*Astl*^*mCherry*^) using fluorescently tagged *lens culinaris* agglutinin (LCA) or ovastacin^mCherry^ (Fig. 1a). To determine whether CGs were enriched in the cortex prior to exocytosis, we imaged zona-free *Astl*^*mCherry*^ eggs at single granule resolution. Live confocal microscopy showed accumulation of CGs in the egg cortex (Fig. 1b). Surprisingly, 3D confocal recordings that sampled the cortex of zona-free eggs every 5 s (Fig. 1c, inset; Supplementary Movies 1, 2) revealed that CGs not only co-localized and moved along actin (Fig. 1c, C2), but actin also polymerized and extended into the imaging plane towards a single CG (Fig. 1c, C1; arrowhead). Following attachment, actin retracted and pulled the CG towards an area, which accumulated a new membrane microdomain (Fig. 1c, C1; dashed area). This contrasted with a stationary CG (Fig. 1c, C1; arrow) which appeared near the membrane at the onset of imaging. The proportion of CGs that associated with actin was higher than those without contact suggesting that CGs interact with the cortical actin scaffold for movement (Fig. 1d). The co-localization and associated movement with cortical actin also were confirmed in fixed samples using super-resolution instant structured illumination microscopy iSIM (Fig. 1e, f).

To follow dynamic movement of granules deeper into the cytoplasm of stimulated mouse eggs, we performed rapid 4D iSIM imaging^19,20^. iSIM projections showed enrichment of CGs beneath the plasma membrane (Fig. 1g, inset; Supplementary Movie 3) and individual CGs could be resolved as they approached the plasma membrane (Fig. 1g; circle in lower panels). Detailed analysis of vesicle movement documented more CGs in the cortex where they also resided for a longer time than CGs present deeper in the cytoplasm (Fig. 1h, i). These results indicate that mouse CGs interact with a dynamic actin cytoskeleton in the egg cortex prior to exocytosis.

### MATER anchors CGs in the egg cortex

**Fig. 2:**
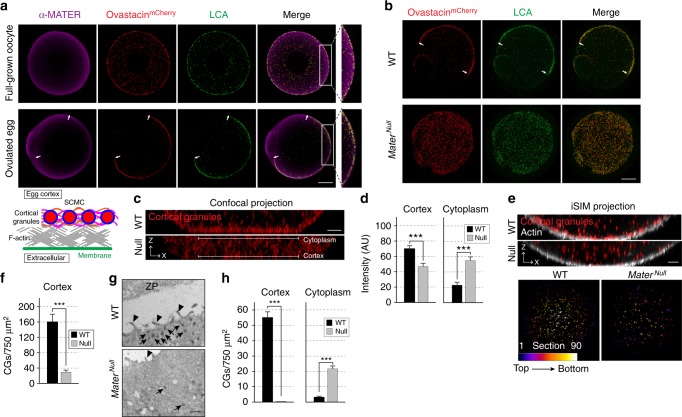
MATER anchors cortical granules in the cortex of mouse eggs. **a** Full-grown, zona-intact oocytes (upper) and ovulated eggs (lower) were collected from ovastacin^mCherry^ (red) transgenic mice, fixed and stained with anti-MATER antibodies (magenta) and LCA-FITC (green) to visualize cortical granules. Confocal cross-sections of z-projections of individual and merged channels include magnified images (right) of the corresponding cortex. Schematic depicts the subcortical maternal complex (SCMC) anchoring cortical granules to the actin cortex, which underlies the plasma membrane. Arrows, extent of cortical granule free zone. Scale bar, 20 μm. **b** As in **a**, but of ovulated wildtype (upper) and *Mater*^*Null*^ (lower) eggs expressing ovastacin^mCherry^. Scale bar, 20 μm. **c** Confocal XZ projections of live wild-type and *Mater*^*Null*^ eggs showing cortical granules (red) after stimulation with SrCl_2_ to trigger cortical granule exocytosis. Scale bar, 5 μm. **d** Quantification of fluorescent intensity in arbitrary units of ovastacin^mCherry^ in cortex and cytoplasmic regions of wild-type (*n* = 21) and *Mater*^*Null*^ eggs (*n* = 15) in **c**. ***p < 0.001 by two-tailed Student’s *t*-test. **e** Stimulated zona-free wild-type and *Mater*^*Null*^ eggs expressing ovastacin^mCherry^ (red) were fixed. F-actin was visualized with phalloidin (gray) prior to imaging with super-resolution iSIM (upper panels, XZ projections). Depth-based pseudo coloring of cortical granules in wild-type and *Mater*^*Null*^ eggs (lower panels, XY projections). Color-scale bar, color and frame number ranging from dark blue (top plane, *Z* = 8 μm) to white (bottom plane, *Z* = 0 μm). Representative iSIM projections are shown. Scale bar, 5 μm. **f** Comparison of cortical granules quantified in the cortex of wild-type (*n* = 10) and *Mater*^*Null*^ eggs (*n* = 6) in **e**. ***p < 0.001 by two-tailed Student’s *t*-test. **g** Transmission electron microscopy of wild-type and *Mater*^*Null*^ eggs. Arrows, cortical granules; arrowhead, microvilli; ZP, zona pellucida. Scale bar, 2 μm. **h** Quantitative comparison of cortical granules in the cortex and cytoplasm of electron micrographs from wild-type (*n* = 11) and *Mater*^*Null*^ eggs (*n* = 12). ***p < 0.001 by two-tailed Student’s *t*-test. Error bar = s.e.m. Source data are provided as a Source Data file

We used *Astl*^*mCherry*^ female mice and antibodies to MATER (official gene name, *Nrlp5*) to confirm the peripheral location of the SCMC and CGs in fully grown oocytes and ovulated eggs (Fig. 2a). We then established *Mater*^*Null*^; *Astl*^*mCherry*^ mice in which CGs did not preferentially accumulate in the cortex of eggs but were scattered throughout the cytoplasm (Fig. 2b). This striking redistribution was quantified by live confocal microscopy of stimulated eggs with >2-fold increase in cytoplasmic fluorescence intensity of mCherry-labeled CGs in the *Mater*^*Null*^ background (Fig. 2c, d). Similar observations were obtained with super-resolution iSIM on fixed samples that documented a >4-fold decrease in the number of CGs in the cortex of *Mater*^*Null*^ eggs (Fig. 2e, f). These findings were confirmed by electron microscopy in which CGs were nearly absent in the cortex, but present in the cytoplasm of *Mater*^*Null*^ eggs, whereas the opposite distribution was observed in wild-type eggs (Fig. 2g, h). Thus, anchoring CGs in the egg cortex requires the presence of maternal MATER.

### Biphasic CG trafficking to the plasma membrane

**Fig. 3:**
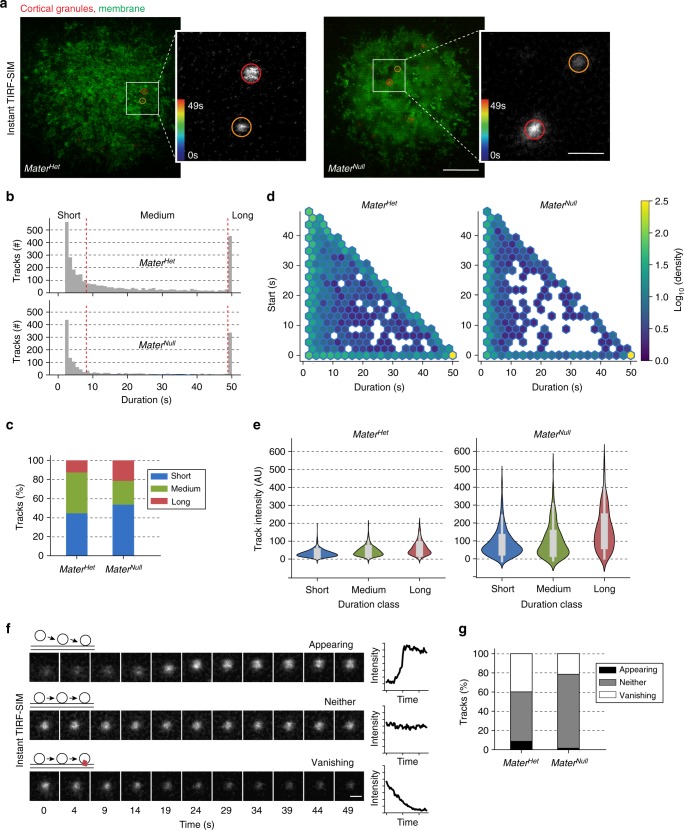
Biphasic cortical granule trafficking to the plasma membrane. **a** Live, instant TIRF-SIM imaging of cortical granules during exocytosis at the plasma membrane. Zona-free *Mater*^*Het*^ (left) and *Mater*^*Null*^ (right) eggs expressing ovastacin^mCherry^ (red) were stimulated to exocytose and imaged in the presence of the membrane dye CellMask (green) at 1 s intervals (×50) (see corresponding Supplementary Movies 4,5). Two representative granules (inset) in circles that are color-coded for duration in seconds (color bar, lower left). Scale bar, 5 μm and 1 μm (inset). **b** Histograms comparing duration of cortical granule tracks in stimulated *Mater*^*Het*^ (top; *n* = 3175) and *Mater*^*Null*^ (bottom; *n* = 1511) eggs. Bimodal distribution with one peak representing short tracks (<8 s) and another peak for long tracks (49 s). Tracks between the two peaks were medium in duration. **c** Stacked column charts of the percentage of the three classes in **b**. **d** Hexbin density plot comparing distributions of cortical granule tracks in stimulated *Mater*^*Het*^ (left; *n* = 3175) and *Mater*^*Null*^ (right; *n* = 1511) eggs. *Y*-axis reflects the start time of an individual track in the 49 s imaging period. A large proportion of long tracks persistent over the entire observation period from 0 to 49 s (yellow hexbin, lower right). Green hexbins vertically represent short tracks that appear and disappear throughout the observation period. Hexbins along the bottom and diagonally represent longer tracks that start or stop within the observation window. **e** Violin plots show fluorescent intensities (arbitrary units) of short-, medium-, and long-duration tracks averaged over the length of each track in stimulated *Mater*^*Het*^ (left) and *Mater*^*Null*^ (right) eggs. The plot boundary captures all data points with the width reflecting the relative kernel density. Within the plot center, the thin and thick gray lines indicate 95% confidence and interquartile range, respectively. **f** Representative examples of enlarged single frames from live instant TIRF-SIM showing single cortical granules classified as appearing, neither, and vanishing together with corresponding intensity plots to the right. **g** Stacked column charts show the percentage of long-duration tracks classified as appearing, neither, or vanishing, based on the intensity profile plot, in stimulated *Mater*^*Het*^ (*n* = 402) and *Mater*^*Null*^ eggs (*n* = 325)

Instant TIRF-SIM images were acquired every 1 s over 50 time points in zona-free mouse eggs to observe the motion of 4686 CGs in zona-free *Mater*^*Het*^ and *Mater*^*Null*^ eggs at the plasma membrane (Fig. 3a; insets; Supplementary Movies 4,5). The imaging rate was sufficient to track the movement and identification of three fluorescent subpopulations based on the duration of their tracks (Fig. 3b). We noted a strong bimodal distribution in *Mater*^*Het*^ eggs with one peak representing short tracks (<8 s) and another peak for long tracks, in which granules were already present at the starting frame and still observed at the last frame of the experiment (49 s). We classified the duration of the tracks between the two peaks as medium. Although short-, medium-, and long-duration granules were present in *Mater*^*Null*^ eggs, the absolute number of CGs (total: 1511) at the plasma membrane was lower (Fig. 3b). This was anticipated based on our earlier observation that the number of granules anchored to the cortex was reduced in *Mater*^*Null*^ eggs (Fig. 2).

Our particle tracking analysis uncovered specific differences in the three subpopulations. As a percentage, *Mater*^*Null*^ eggs not only contained more short and long tracks (Fig. 3c; chi-squared test, *p* < 2.2e^−16^), but they were also substantially depleted in tracks of medium duration when visualized using a hexbin density plot (Fig. 3d, diagonal and bottom middle of plots). In addition, short-duration granules moved faster than their medium- or long-duration counterparts in the absence of MATER (Supplementary Fig. 3a, b). We further interrogated the mean fluorescent track intensities across the three subpopulations and did not observe significant differences in the track intensities of short-, medium-, and long-duration granules in *Mater*^*Het*^ eggs. Notably, track intensities were substantially higher in all three populations in stimulated *Mater*^*Null*^ eggs (Fig. 3e; Supplementary Fig. 4). Whereas the absence of MATER does not appear to affect trafficking to the plasma membrane from any subpopulation, our data indicates that the interaction of granules with the plasma membrane and the subsequent release of ovastacin is impaired.

Next, we analyzed the track intensity of only long-duration granules. We identified individual appearing granules in *Mater*^*Het*^ eggs that moved into focus at the plasma membrane and became brighter (Fig. 3f) suggesting that sustained secretion requires arrival of new vesicles at the plasma membrane, which are rendered fusion-competent^21^. Strikingly, appearing vesicles were nearly absent in *Mater*^*Null*^ eggs indicating that recruitment of new granules to the membrane was impaired (Fig. 3g; Supplementary Fig. 4). We also identified vanishing granules, where the fluorescent intensity decayed (Fig. 3f, g). Since instant TIRF-SIM does not indicate directionality, we cannot determine whether the disappearance of granule fluorescence was due to exocytosis or retreat into the cell interior. However, having established that the track intensity of long-duration granules was significantly higher in *Mater*^*Null*^ eggs due to impaired interactions at the plasma membrane, we favor the model that most of the vanishing granules are undergoing exocytosis and noted fewer vanishing granules in *Mater*^*Null*^ eggs (Fig. 3g; Supplementary Fig. 4). These differences suggest that exocytosis could be delayed in the absence of MATER.

We also clustered tracks into a third category, which neither conformed to appearing nor vanishing profiles. This neither category contained a range of intensity profiles (Fig. 3g; Supplementary Fig. 4), but the fluorescent intensity remained roughly constant or varied enough that no clear pattern was observed (Fig. 3f). Interestingly, this category of granules was predominantly observed in *Mater*^*Null*^ eggs suggesting that it could further contribute to a delay in exocytosis. Thus, particle tracking at high spatiotemporal resolution with instant TIRF-SIM in combination with computational analysis showed that anchoring CGs critically affects their trafficking from the cortex to the plasma membrane in mouse eggs.

### Non-muscle myosin IIA associates with CGs

**Fig. 4:**
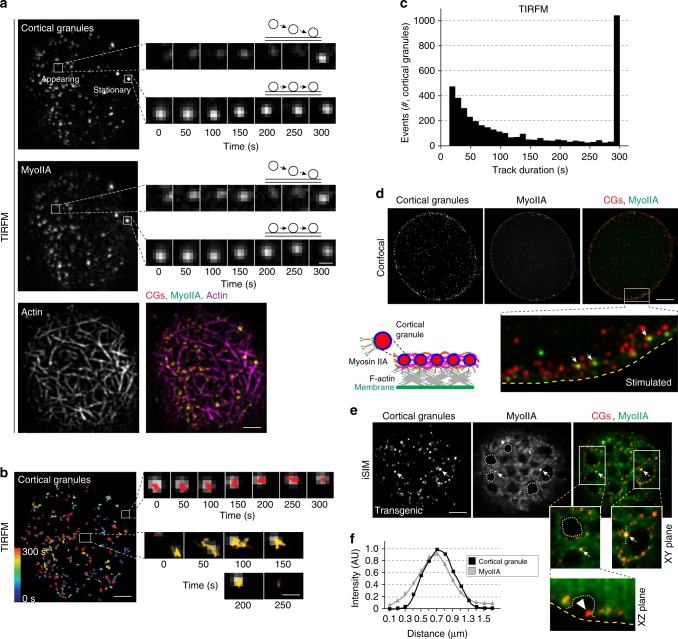
Non-muscle myosin IIA associates with mouse cortical granules. **a** Eggs expressing transgenic ovastacin^mCherry^ and knock-in myoIIA^EGFP^ were sampled every 10 s for 5 min in the presence of the fluorogenic probe SiR-actin for F-actin at the plasma membrane using diffraction-limited TIRFM (see corresponding Supplementary Movie 6). A representative TIRF image showing cortical granules (upper left; red in merge), myoIIA (middle left; green in merge), actin (lower left; magenta in merge) and merge (lower right). Scale bar, 5 μm. Enlarged single frames of live TIRFM showing an approaching and stationary cortical granule at indicated time points: ovastacin^mCherry^ (upper right) and myoIIA^EGFP^ (middle right). Scale bar, 1 μm. **b** First frame from TIRFM time-lapse series overlaid with temporal, color-coded vesicle tracks. Color-scale bar, color and time point ranging from dark blue (*t* = 0 s) to red (*t* = 300 s). Enlarged views of two-colored tracks corresponding to the insets at indicated time points. Scale bars, 5 μm (left), 1 μm (right). **c** Histogram of bimodal distribution of cortical granule tracks (*n* = 5496) during live-imaging (300 s) in stimulated ovastacin^mCherry^ eggs. Source data are provided as a Source Data file. **d** Fixed zona-free mouse eggs expressing ovastacin^mCherry^ and myoIIA^EGFP^ were stimulated for 15 min to trigger exocytosis and imaged with confocal microscopy. Representative cross-sections of confocal z-projections are shown with an enlarged view of the white inset (below). Arrows, EGFP puncta, near single cortical granules; dashed yellow line, plasma membrane. Schematic shows the association of myoIIA with cortical granules. Scale bar, 20 μm. **e** Representative iSIM projection of egg cortex showing cortical granules (red) and myoIIA^EGFP^ (green) with a magnified view (below) of two regions of interest (white insets) in XY and XZ planes. Arrows, single cortical granules overlapping with EGFP puncta; white dashed lines, regions of void EGFP; arrowhead, single cortical granule; yellow dashed line, plasma membrane. Scale bar, 5 μm. **f** Profile plot of dashed line in magnified **e** (inset of right panel) indicating peaks in mCherry (cortical granules) and EGFP channel of myoIIA

We determined that fluorescent myoIIA in transgenic mice^22^ was present in the cortex of fixed, phalloidin-stained fully-grown oocytes and stimulated eggs (Supplementary Fig. 5a). To investigate the possibility that myoIIA was recruited onto CGs, we generated mice carrying both the fluorescent reporter knock-in *MyoIIA*^*EGFP*^ and *Astl*^*mCherry*^ transgenes. Acquiring TIRF images every 5 s over 5 min was sufficient to follow CGs for an extended period (Fig. 4a; Supplementary Fig. 1a–d; Supplementary Movie 6). Intriguingly, myoIIA^EGFP^ associated with two populations of granules: appearing and stationary. As previously documented with instant TIRF-SIM, we noted appearing granules moving into the TIRF field over time and becoming brighter. In contrast, the brightness did not change in stationary granules, which were present from the first to the last frame of the experiment (Fig. 4a). Tracking the motion of 5496 CGs and plotting their bimodal distribution over the 5-min imaging period showed that 1033 (~20%) were stationary (Fig. 4b, c).

Confocal sections of fixed eggs not only showed accumulation of myoIIA^EGFP^ and ovastacin^mCherry^ in the cortex, but also diffraction-limited puncta of EGFP-labeled myoIIA, some of which appeared near ovastacin^mCherry^-labeled CGs (Fig. 4d; inset; arrows). To spatially resolve the two signals, we turned to super-resolution iSIM. Notably, myoIIA^EGFP^ diffusively localized around EGFP-void areas 15 min post stimulation (Fig. 4e; dashed area). However, we also observed a preferential enrichment of myoIIA^EGFP^ as a set of puncta (Fig. 4e; arrows). The spatial resolution of iSIM indicated that only single punctum marking EGPF-labeled myoIIA co-localized with mCherry-labeled CGs (Fig. 4e, f). The data must be interpreted cautiously, however, because at iSIM resolution, a single punctum could contain more than one molecule. Interestingly, we also detected the presence of CGs inside EGFP-void areas (Fig. 4e; XZ plane; arrowhead). As the motor domain in the head portion of the myoIIA molecule contains a well-characterized actin-binding site^23,24^, we reasoned that myoIIA^EGFP^ puncta were recruited to newly assembled actin. Immunostaining of myoIIA^EGFP^ expressing eggs with phalloidin confirmed that single myoIIA^EGFP^ puncta overlapped with actin-rich foci (Supplementary Fig. 5b, c). Altogether, these results suggest that non-muscle myoIIA associates with mouse CGs trafficking to the plasma membrane for exocytosis.

### Clearance of cortical actin prior to exocytosis

**Fig. 5:**
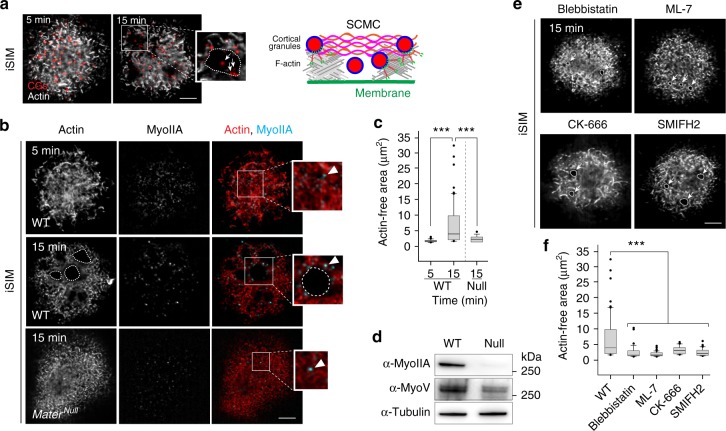
Clearance of egg cortex during cortical granule exocytosis. **a** Zona-free ovastacin^mCherry^ eggs were stimulated to exocytose and fixed after 5 and 15 min followed by immunostaining with phalloidin to label F-actin. Representative iSIM maximum intensity projection of egg cortex showing cortical granules (red) and actin (gray) with magnified view of actin-free area. Arrows, cortical granule; white dashed line, actin-free area. Schematic (right) shows clearance of cortical actin during exocytosis and the appearance of cortical granules inside cleared actin sites. Scale bar, 5 μm. **b** Zona-free wild-type and *Mater*^*Null*^ eggs were stimulated to exocytose and fixed after 5 and 15 min followed by immunostaining with phalloidin (red) and antibody specific to myoIIA (cyan). Representative iSIM projection of the egg cortex with magnified views of regions of interest (white insets). Arrowhead, myoIIA puncta; white dashed lines, actin-free area. Scale bar, 5 μm. **c** Quantification of actin-free areas in the cortex of fixed stimulated wild-type eggs after 5 (*n*, 20) and 15 min (*n* = 78), and *Mater*^*Null*^ eggs (*n* = 55) acquired by iSIM. Box plot includes the mean (horizontal line) and data between the 25th and 75th percentile. Error bars indicate the 90th and 10th percentiles. ***p < 0.001 by two-tailed Student’s *t*-test. The experiment reflects three biologically independent replicates. **d** Immunoblot of egg (60) lysates isolated from wild-type or *Mater*^*Null*^ female mice and probed with antibodies specific to myoIIA and myosin V (MyoV). Molecular mass, right. **e** Zona-free wild-type eggs were stimulated to exocytose in the presence of myosin II inhibitors blebbistatin and ML-7 as well as actin-nucleation inhibitors CK-666 and SMIFH2. Eggs were fixed after 15 min followed by immunostaining with phalloidin (gray). White dashed lines, actin-free area. Scale bar, 5 μm. **f** Quantification of actin-free areas in the cortex of wild-type eggs (*n* = 78) and eggs treated with blebbistatin (*n* = 35), ML-7 (*n* = 52), CK-666 (*n* = 44), and SMIFH2 (*n* = 62) acquired by iSIM. Box plot includes the mean (horizontal line) and data between the 25th and 75th percentile. Error bars indicate the 90th and 10th percentiles. ***p < 0.001 by two-tailed Student’s *t*-test. The experiment reflects three biologically independent replicates. Source data are provided as a Source Data file

Cortical actin presents a barrier that cells must first clear before subcellular vesicles can fuse with the plasma membrane^25^. To determine whether myoIIA^EGFP^-void areas correlated with the clearance of cortical actin, we analyzed fixed, ovastacin^mCherry^ eggs after stimulation for 5 or 15 min using iSIM. Whereas a mostly dense cortical actin scaffold was present after 5 min of stimulation (Fig. 5a), we observed prominent actin-free areas after 15 min (Fig. 5a; inset, dashed area). Consistent with the presence of CGs in myoIIA^EGFP^-void areas (Fig. 4e), we also detected CGs inside actin-free areas (Fig. 5a; inset, arrows) suggesting that local clearance of the cortical actomyosin network could provide access of CGs to the plasma membrane for exocytosis (Fig. 5a; schematic). In contrast, a diffuse and dense cortical actin meshwork persisted in *Mater*^*Null*^ eggs even after 15 min of stimulation indicating that the absence of MATER not only impairs CG anchoring at the egg cortex, but also cortical actin turnover associated with exocytosis in mouse eggs (Fig. 5b, c).

We hypothesized that myoIIA mediated contraction leads to actin clearance. Intrigued, however, by our earlier observation that actin clearance was impaired in the absence of MATER, we first performed immunoblot analysis in unstimulated wild-type and *Mater*^*Null*^ eggs using an isoform-specific myoIIA antibody. Strikingly, immunoblots detected intact myoIIA protein in wildtype, but only small amounts in *Mater*^*Null*^ eggs (Fig. 5d). Myosin V, implicated in CG trafficking^7^, was also decreased in *Mater*^*Null*^ eggs indicating that more than one class of myosins is affected in the absence of MATER. To rule out the possibility that low protein expression levels in mutant eggs masked the detection of sub-diffractive myoIIA, we also imaged the cortex using iSIM and observed myoIIA puncta in stimulated *Mater*^*Null*^ eggs (Fig. 5b) indicating the presence of myoIIA, albeit at low levels.

Blebbistatin inhibits myoIIA motor ATPase activity, the small molecule inhibitor ML-7 targets the myosin light chain kinase (MLCK), which is responsible for regulatory light chain (RLC) phosphorylation and myoIIA filament assembly^26,27^. In contrast, actin dynamics are largely driven by Arp2/3- and formin-dependent actin nucleation, which are inhibited by CK666 and SMIFH2, respectively, and myoIIA-dependent contractile forces^28,29^. Stimulation of zona-free eggs in the presence of myoIIA and actin inhibitors for 15 min led to repression of actin clearance (Fig. 5e, f). Together, these results argue that cortical actin is locally cleared in mouse eggs prior to exocytosis. This process is not only dependent on maternal MATER, but also on myoIIA activity and initial actin nucleation.

### Impaired trafficking delays exocytosis and causes polyspermy

**Fig. 6:**
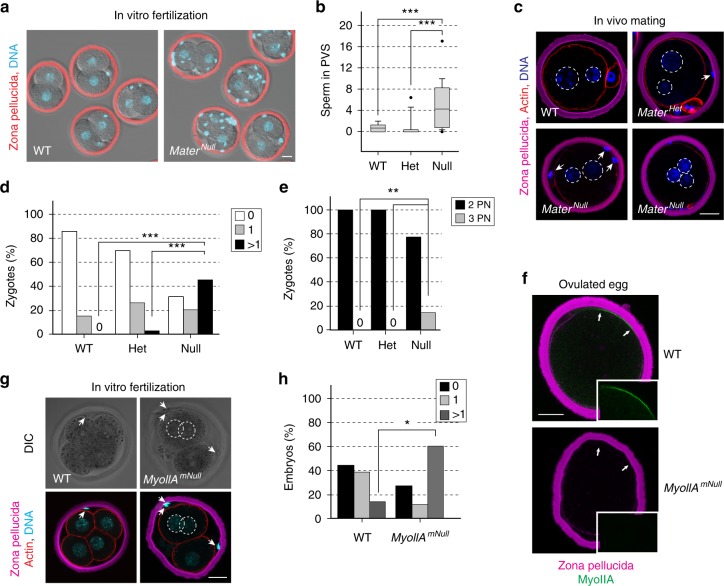
Impaired trafficking leads to delayed exocytosis and polyspermy. **a** Ovulated eggs from wild-type (left) and *Mater*^*Null*^ (right) female mice were inseminated, cultured to two-cell embryos and stained with WGA-Alexa Fluor 633 lectin (red, zona pellucida) and Hoechst 33342 (blue, DNA). Scale bar, 20 μm. **b** The number of sperm in the perivitelline space (PVS) for each genotype (9–36 two-cell embryos from three biologically independent samples). Box plot includes the mean (horizontal line) and data between the 25th and 75th percentile. Error bars indicate the 90th and 10th percentiles; outliers are indicated by dots. ***p < 0.001 by two-tailed Student’s *t*-test. **c** In vivo fertilized *Mater*^*Het*^ and *Mater*^*Null*^ zygotes were fixed for staining with WGA-Alexa Fluor 633 (zona pellucida), phalloidin (F-actin) and DAPI (DNA). Arrows, supernumerary sperm in the PVS; pronuclei enclosed by dashed line. Scale bar, 20 μm. **d** Quantification of sperm in the PVS from **c** was determined for each genotype (13–60 embryos from three biological samples). ***p < 0.001 by chi-squared test. **e** Quantification of pronuclei in embryos from **c**. **p < 0.01 by chi-squared test. **f** Ovulated eggs from wildtype (upper) and *MyoIIA*^*mNull*^ (lower) were immunostained with WGA-Alexa Fluor 633 (magenta) and anti-MyoIIA (green). Representative confocal cross sections of z-projections are shown with a magnified view (inset) of the myoIIA region indicated by arrows. Scale bar, 20 μm. **g** After in vitro insemination, wild-type and *MyoIIA*^*mNull*^ eggs were cultured to the 4-cell stage and fixed for staining with WGA-Alexa Fluor 633 (zona pellucida), phalloidin (F-actin) and DAPI (DNA). Arrows, supernumerary sperm in the perivitelline space; dashed lines, nuclei. Scale bar, 20 μm. **h** The number of sperm in the PVS for each genotype in **g** (15–18 embryos from two biologically independent samples). ***p < 0.001 by chi-squared test. Source data are provided as a Source Data file

To determine the biological significance of impaired CG trafficking and actin clearance for the zona pellucida block to polyspermy, we performed in vitro fertilization of *Mater*^*Null*^ and wild-type eggs. We noticed significantly more sperm in the perivitelline space of two-cell embryos (Fig. 6a, b; 5.4 ± 0.69 s.e.m.; Student’s *t*-test, *p* < 0.001) after fertilization of eggs from *Mater*^*Null*^ females indicating a decrease in the zona block to penetration. This deprecation of the post-fertilization zona block was also observed in vivo (Fig. 6c) where more than 60% of fertilized zygotes collected from *Mater*^*Null*^ females had one or more sperm in the perivitelline space (Fig. 6c, d; arrows; 1.82 ± 0.25 s.e.m.; chi-squared test, *p* < 0.001). The increased frequency of additional sperm in the perivitelline space was also detected in two-cell embryos derived from *Mater*^*Null*^ females (Supplementary Fig. 6a, b).

More striking, the number of zygotes with three pronuclei collected from *Mater*^*Null*^ females after in vivo mating increased to 15%. Consequently, one of the blastomeres in two-cell embryos from *Mater*^*Null*^ females contained two nuclei instead of one (Fig. 6e; Supplementary Fig. 6c). To follow the kinetics of CG exocytosis, we acquired confocal z-stacks of stimulated live whole mount eggs expressing ovastacin^mCherry^ at 10-min intervals. The intensity of ovastacin^mCherry^ fluorescence in the cortex of eggs heterozygous for the *Mater* null allele declined sharply 10–20 min after stimulation. In contrast, in eggs homozygous of the *Mater* null allele, there was an increase in cortical fluorescence 60 min after stimulation, presumably due to the continued translocation of CGs from the interior to the periphery of the egg (Supplementary Fig. 6d, e; Supplementary Movies 7, 8). The delayed CG exocytosis in *Mater*^*Null*^ eggs significantly affected ZP2 cleavage which remained intact 30 min after stimulation (Supplementary Fig. 6f, see Supplementary Fig. 8 for uncropped images), thus allowing additional sperm to bind and penetrate the zona matrix. A similar effect on the post-fertilization block to polyspermy was not observed in the absence of FLOPED (official gene name, *Ooep*) another component of the SCMC (Supplementary Fig. 7).

Due to maternally imposed embryonic lethality that precludes progression beyond 2–4 cell, *Mater*^*Null*^ mice are infertile in vivo.^12^ To determine the effect of myoIIA on in vivo fertility, *MyoIIA*^*Flox/Flox*^ and egg-specific *(Tg)Zp3-Cre* mice were crossed to establish a mouse line (*MyoIIA*^*mNull/mNull*^) with an egg-specific (maternal) conditional deletion of the gene (Fig. 6f; Supplementary Fig. 6g). These female mice had smaller litters (3.3 ± 1.7) compared to *MyoIIA*^*Flox/Flox*^ female mice (7.0 ± 2.4). After in vitro fertilization, significantly more sperm were observed in the perivitelline space of embryos derived from *MyoIIA*^*mNull/mNull*^ females (Fig. 6g, h; arrows; 4.27 ± 1.34 s.e.m.; chi-squared test, *p* < 0.001) indicating a decrease in the zona block to penetration which could account for the lower fertility of *MyoIIA*^*mNull/mNull*^ mice. Taken together, these results document that MATER, a component of the SCMC, and non-muscle myosin IIA participates in CG trafficking and timely exocytosis to prevent polyspermy in mouse eggs.

## Discussion

**Fig. 7:**
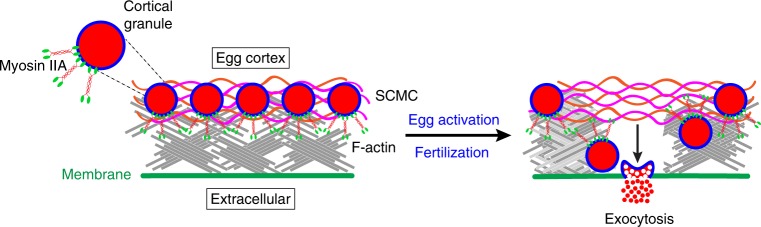
Model of cortical granule dynamics at the cortex-plasma membrane interface to prevent polyspermy in mouse eggs. MATER anchors cortical granules in the cortex underlying the egg’s plasma membrane where they associate with non-muscle myosin IIA, a motor protein. During fertilization and egg activation, cortical actin is cleared and cortical granules traffic to the plasma membrane. Fusion of cortical granules at the plasma membrane releases their contents, including zinc and the metalloendopeptidase ovastacin, into the extracellular space. These cortical granule contents prevent sperm penetration of the zona matrix and cleave the zona pellucida protein ZP2 to ensure monospermic fertilization

Three post-fertilization blocks to gamete interaction have evolved in mammals to prevent polyspermy that is embryonic lethal. In mice, the first two occur rapidly after fertilization and prevent additional sperm from fusing with the egg plasma membrane or penetrating the surrounding extracellular zona pellucida^18,30,31^. The third and definitive block prevents sperm from even binding to the surface of the zona matrix and depends on egg CG exocytosis and the resultant cleavage of ZP2. Using diffraction-limited and super-resolution imaging of individual CGs fluorescently stained with ovastacin^mCherry^ in transgenic mice, we have investigated CG dynamics at the cortex-plasma membrane interface that prevents polyspermy in mouse eggs. Our data support a model in which MATER is present in the cortex underlying the egg’s plasma membrane where it anchors CGs which are associated with non-muscle myosin IIA. During fertilization and egg activation, cortical actin is cleared and myoIIA is required for CGs to traffic to the plasma membrane prior to exocytosis (Fig. 7).

The SCMC is formed in the subcortex of oocytes during oogenesis and is composed of at least five components including MATER and FLOPED^12–16^. While investigating a possible role in CG biology, we determined that MATER was required to position CGs in the periphery near the egg plasma membrane. The SCMC is symmetrically present in MII eggs^13^ including a CG free region that is established by an actin cap^32^. Curiously, although MATER is not present in the subcortex in the absence of FLOPED^13^, we did not observe displacement of CGs in eggs derived from homozygous *Floped*^*Null*^ female mice. Like the other SCMC members, MATER has motifs associated with protein-protein interaction, specifically 13 leucine-rich repeats^33^ near its carboxyl terminus. Whether cortical anchoring of CGs by MATER is facilitated by direct or indirect interaction(s) with proteins present on the vesicular membrane is currently not known. However, MATER appears to be necessary, but not sufficient, for positioning CGs in the subcortex of mouse eggs.

In the present study, we document a dense actin meshwork underlying the egg plasma membrane where CGs are anchored. Pharmacological disruption of cortical actin in mouse eggs results in an increased appearance of granules near the plasma membrane prior to exocytosis which supports a model whereby cortical actin acts as a barrier to granule docking. A similar model has been proposed for bi-phasic insulin-granule exocytosis^34,35^ in which F-actin is organized as a dense web beneath the plasma membrane to impede access of insulin-containing granules to the cell surface^36,37^. The first rapid phase involves membrane fusion of a limited pool of release-ready insulin granules already present at the plasma membrane^38,39^. Insulin granules that are deeper within the cell and trafficked to the cell periphery during the second phase secretion slowly replenish this pool^39^, similar to what is observed with CGs. In our model, long-duration vesicles, which are tethered to the plasma membrane, could be required for a slow and sustained release of cargo molecules, whereas short-duration vesicles likely interact transiently with the plasma membrane for a more rapid release as cortical actin is cleared.

Whereas biphasic trafficking is not affected in the absence of MATER, the interaction of long-duration granules is significantly impaired. Furthermore, we uncover an unexpected role for CGs of medium-duration. The strong decrease of medium-duration granules and the delay in exocytosis in *Mater*^*Null*^ eggs suggests that this subpopulation of CGs is directly or indirectly required for one or more steps of secretion in mouse eggs, either by priming long-duration vesicles for the second phase of secretion by an unknown mechanism or coordinating timely clearance of cortical actin as well as trafficking of short-duration vesicles to the plasma membrane.

Alternatively, one or two myosin II isoforms could generate pulsatile contractile forces on the cortex, which would drive clearance of cortical actin during exocytosis^40–42^. Our finding that clearance of cortical actin is repressed in the presence of myosin inhibitors supports the concept that ATPase activity and activation of myoIIA are required to disassemble cortical actin. We provide genetic evidence in *Mater*^*Null*^ eggs on the importance of myoIIA expression for actin clearance during exocytosis observing strongly reduced myoIIA levels, which could also impair pulsatile contractile forces. In addition to myosin II, myosin V was also reduced in *Mater*^*Null*^ eggs. Importantly, myosin V has been demonstrated to associate with insulin-containing granules and CGs^7,43^, illustrating the requirement of the myosin motors behind granule movement to the cell periphery and secretion.

Unlike other secretory organelles, CGs are not renewed or maintained in the cytoplasm once released at the plasma membrane. Even though instant TIRF-SIM does not indicate directionality, the disappearance of single granule fluorescence appears to be an example of exocytosis rather their retreat into the cell interior and rejoining of the cytoplasmic pool. This conclusion is furthermore supported in *Mater*^*Null*^ eggs in which exocytosis is delayed containing fewer disappearing, vanishing granules.

After fertilization, CGs release ovastacin, a metalloendopeptidase that cleaves ZP2 and precludes subsequent sperm binding to the zona pellucida^17^. As post-fertilization cleavage of ZP2 proceeds with slow kinetics (10–20 min) after in vitro stimulation, the coordination of actin clearance and exocytosis appears to be well adapted to the small size of mouse CGs and the regulated release of their contents. In the absence of MATER, supernumerary sperm accumulate in the perivitelline space between the egg plasma membrane and the inner aspect of the extracellular zona pellucida matrix. This is associated with delayed release of ovastacin and cleavage of ZP2, and is in accord with earlier studies in which genetic ablation of ovastacin prevents the normal post-fertilization block to sperm binding and penetration of the zona matrix^18^. The molecular basis of the post-fertilization block to gamete fusion remains indeterminate, but is reported to be independent of CG exocytosis^30,44^ and can be bypassed by intracellular sperm injection^31,45^. The relatively low level of polyspermy that we observe in *Mater*^*Null*^ (15%) is more striking in *MyoIIA*^*mNull*^ eggs which raises the possibility that the trafficking of CGs to the plasma membrane plays a role, either directly or indirectly, in the membrane block to gamete fusion.

## Methods

### Ethical compliance and source of reagents

Mice were maintained in compliance with the guidelines of the Animal Care and Use Committee of the National Institutes of Health under a Division of Intramural Research, NIDDK-approved animal study protocol. Unless otherwise noted, chemical and other reagents were obtained from Sigma-Aldrich. See Supplementary Table 2 for parameters of imaging experiments.

### Mice and genotyping

*Mater*^*Null*^ (^12^) and *Floped*^*Null*^ (^13^) mice expressing ovastacin^mCherry^ were generated by crossing the KO lines to transgenic *Astl*^*mCherry*^ mice^32^. Transgenic *Astl*^*mCherry*^ mice were also crossed to mice expressing EGFP-tagged non-muscle myosin IIA (*MyoIIA*^*EGFP*^)^22,46^. Conditional *MyosinIIA*^*mNull*^ (*MyoIIA*^*mNull*^) mice were generated by crossing homozygous female *MyosinIIA*^*Flox/Flox*^ mice to homozygous floxed males positive for *Tg* (*Zp3-Cre*)^47,48^. For genotyping, tail tips of mice were lysed, and DNA was extracted using DNeasy Blood & Tissue Kit according to the manufacturer’s protocol (Qiagen). EmeraldAmp GT CR Master Mix (Takara Bio USA) and gene specific primers (Supplementary Table 1) were used to amplify specific DNA fragments. PCR was performed with annealing temperatures of 58 °C and 30 cycles (for *Ovastacin*^*mCherry*^), 58 °C and 28 cycles (for *Mater*), 55 °C and 30 cycles (for *Floped*), 58 °C and 40 cycles (for *MyosinIIA*^*EGFP*^), 60 °C and 30 cycles (for *Cd9*), 58 °C and 35 cycles (for *MyosinIIA*^*Flox*^), 51.7 °C and 35 cycles (for *Zp3-Cre*).

### Preparation of eggs and embryos for microscopy

Eggs and embryos were collected from oviducts of 8–12 week-old females that had been induced to ovulate with 5 IU equine chorionic gonadotropin (eCG) followed by 5 IU human chorionic gonadotropin (hCG) 48 h later^13,49^. Eggs were collected approximately 16 h after administration of hCG, placed in EmbryoMax M2 medium containing hyaluronidase (Millipore) until cumulus cells dispersed. For all experiments involving imaging of zona-free eggs, the zona pellucida was removed by rapid (~30 s) treatment with EmbryoMax Tyrode’s solution (Millipore). To induce exocytosis, eggs were parthenogenetically activated in CaCl_2_-free M16 medium supplemented with 5 mM SrCl_2_ at 37 °C in 5% CO_2_. In all experiments involving cytoskeletal inhibitors, zona-free eggs were parthenogenetically activated for 15 min in the presence of 100 µM blebbistatin (EMD Millipore), 40 µM ML-7, 100 µM CK666, 10 µM SMIFH2 or DMSO as a control.

For in vitro fertilization, eggs were incubated in human tubal fluid medium (Zenith Biotech) for 6 h at 37 °C with fresh motile sperm (5 × 10^5^ ml^−1^) which had been capacitated for 2 h at 37 °C. Zygotes and two-cell embryos were also obtained from females hormonally induced to ovulate followed by in vivo mating with males proven to be fertile. The presence of a copulation plug on the following day was used to confirm successful mating. Pronuclear-staged zygotes and two-cell embryos were isolated from oviducts and collected in M2 medium^49^.

### Antibodies and immunofluorescence

Zona-intact eggs and early embryos were fixed for 20 min at 37 °C in 100 mM PIPES (pH 6.9), 5 mM MgCl_2_, 2 mM EGTA (pH 6.9), 2% paraformaldehyde, 0.5 % Triton X-100, and 50% deuterium oxide. Zona-free eggs were fixed as described, but Triton X-100 was omitted. Following fixation, eggs/embryos were washed in PBS containing 3 mg ml^−1^ polyvinylpyrolidone (PBS/PVP), permeabilized for 30 min in PBS/PVP containing 0.25% Triton X-100 and incubated in blocking buffer (0.1% BSA, 0.01% Tween 20 and 2% donkey serum) prior to immunofluorescence^50^. Eggs/embryos were incubated with primary antibodies to MATER^51^ (1:500) and FLOPED^13^ (1:200) overnight at 4 °C, washed with blocking buffer (three times, 10 min each), and incubated (60 min) with secondary antibody (1:100) followed by staining DNA with Hoechst 33342 (Invitrogen). The rabbit polyclonal antibody against non-muscle myosin IIA (kindly provided by Dr. Robert Adelstein, NHLBI, NIH) was used at 1:100 dilution. CGs were immunostained with the conjugated lectin LCA-FITC (Cosmo Bio USA) at a 1:100 dilution.

### Fixed and live confocal microscopy

Images were acquired with a Zeiss LSM 780 confocal microscope equipped with a Stable Z heating system (Bioptechs) and a 40x C-Apochromat 1.2 NA water-immersion objective lens. For live imaging, zona-free eggs were mounted on poly-L-lysine coated Bioptechs Delta-T culture dishes with pre-warmed M2 or CaCl_2_ -free M16 medium at 37 °C in 5% CO_2_. Typically, cells were first imaged by taking 6–8 μm *z*-stacks with 0.2 μm axial steps near the coverslip followed by time lapse acquisition in a single focal plane close to the coverslip every 5 s for up to 5 min. Images were then deconvolved using Huygens Essential Software version 4.0 (Scientific Volume Imaging). A theoretical point-spread function was based on microscope parameters, model confocal parameters established by Huygens and the Classic Maximum Likelihood Estimation algorithm was used to restore images. Sampling intervals were set manually to the actual experimental values, together with refractive indices and excitation/emission wavelengths.

### Total internal reflection fluorescence microscopy (TIRFM)

For TIRF imaging of CGs, zona-free eggs were mounted as described for confocal microscopy. Oocytes were excited using a 100 mW 488-nm laser introduced at the appropriate incident angle through the TIRF-slider (Zeiss) and ×63 objective (Zeiss, 1.46 NA Oil, alpha Plan-Apochromat). Images were acquired with a digital Evolve 512 EMCCD camera (Photometrics, Tucson, AZ, USA) controlled with Zeiss Zen 2 software. The size of each pixel was 254 × 254 nm. To observe CG motion ~250 nm near the plasma membrane in mouse eggs, cells were imaged every 10 s for 5 min. Images were taken with an exposure time of 200 ms using 4% maximal laser output. Under these experimental conditions, no reduction in fluorescence from photobleaching was observed.

### Instant structured illumination microscopy (iSIM)

Images were acquired with a VT-iSIM Multi-Point Super Resolution Imaging System (VisiTech International) connected to an Olympus microscope body via a regular c-mount that housed objectives (Olympus UPLSAPO 60XS, 1.3 NA Silicone Oil, for live and fixed sample imaging, or 100 × 1.49 NA Oil objective, for fixed zona-free mouse eggs), an automated XY stage (Applied Scientific Instrumentation) and a custom-built heating System to maintain 37 °C for live-imaging experiments. The imaging pixel size for the ×60 and ×100 objective was 107 nm and 64 nm, respectively. For excitation, 488 nm, 561 nm and 642 nm lasers were used. Lasers were combined with an automated 3-position dichroic changer. For live-imaging, zona-free eggs were mounted on poly-L-lysine coated 35 mm glass bottom microwell dishes (MatTek Corporation). Image acquisition was controlled with Meta-Morph software. Images were acquired with an exposure time of 250 ms using 25–40% maximal laser output. The illumination intensity at the back aperture was measured at 1.23 mW, 0.42 mW and 0.26 mW for 488 nm, 561, nm and 642 nm excitation, respectively. *Z*-stacks were captured with 0.2 μm axial steps (30–35 z-sections) for live samples and 0.1 μm axial steps (80–90 z-sections) for fixed samples. Images were subjected to GPU deconvolution^52^ using an ImageJ plugin with a calculated point spread function, destriping and background-subtraction (Microvolution LLC). Although 3D imaging techniques including confocal microscopy subject the mouse eggs to more dose than do TIRF techniques, we did not observe substantial bleaching or phototoxicity in our live experiments, suggesting that these factors did not influence our study. In addition, brightfield inspection of the morphology did not suggest any light-induced effects.

### Live instant TIRF-SIM

Instant TIRF-SIM experiments were conducted on a laboratory assembled instant SIM^19^ that had been modified to provide TIRF illumination^20^. Briefly, the main changes to the original instant SIM were: (1) placement of an annular mask in an upstream plane conjugated to the back focal plane of the objective, thereby removing low-angle, subcritical illumination and ensuring TIRF; and (2) using an ultrahigh numerical aperture (NA) objective lens (Olympus, APON100xHOTTIRF, 1.7 NA) with matched, high-index coverslips (Olympus, 9-U992) to facilitate TIRF. The imaging pixel size was 33.4 nm. 488- and 561-nm lasers were used for illumination, and either notch (Semrock, NF03-488E-25 and NF03-561E-25 notch filters for imaging green and red emission) or bandpass (Semrock, FF03-525 525/50, for imaging green emission) filters were employed to filter fluorescence. Zona-free eggs were mounted on poly-L-lysine coated high-index coverslips and imaged within the evanescent penetration depth of ~120 nm. Exposure times were typically 40–50 ms. Illumination laser power before the objective was measured at 1.0 mW, implying an average intensity (given the 58 μm × 52 µm field of view) of ~33 W/cm^2^. Temperature was maintained at 37 °C using an incubation chamber (Warner Instruments, H301-MINI). The raw images acquired by instant TIRF-SIM were post-processed, including background subtraction and deconvolution, to achieve the full twofold resolution gain relative to conventional widefield fluorescence imaging. Background was usually estimated by averaging 100 images acquired without illumination. For deconvolution, we used the Richardson-Lucy algorithm with an experimental point spread function derived by registering and then averaging twenty 100 nm yellow-green beads. Deconvolution was implemented in MATLAB 2017a (Mathworks). The number of iterations was set to 10 for all images.

To validate these protocols, we imaged zona-free eggs from *Cd9*^*Null*^ female mice. CD9 is a tetraspanin protein that localizes to microvilli of membranes^53^ and *Cd9*^*Null*^ eggs have an altered membrane due to varied length, thickness and density of their microvilli^54^. Projected confocal images detected a >1.5-fold reduction in the fluorescent intensity of *Cd9*^*Null*^ compared to wild-type eggs (Supplementary Fig. 2g, h). This observation suggests an altered cell surface that adversely affects the uptake of membrane dye and was confirmed at higher spatial resolution using instant TIRF-SIM (Supplementary Fig. 2i).

### Live-cell F-actin and plasma membrane labeling

F-actin was labeled with 1 μM of the live-cell Spirochrome probe SiR-actin (Cytoskeleton) which is based on natural actin-binding jasplakinolide. The plasma membrane was labeled with CellMask Green using the manufacturer’s recommended 1x working solution (ThermoFischer Scientific). Zona-free eggs were mounted on poly-L-lysine coated coverslips) with pre-warmed M2 or CaCl_2_ -free M16 medium supplemented with SiR-actin, CellMask Green and incubated at 37 °C in 5% CO_2_ for 30 min prior to imaging. Neither CG exocytosis or fertilization was inhibited under these experimental conditions even with 5 μm SiR-actin (Supplementary Fig. 2j, k).

### Electron microscopy

Super-ovulated eggs from wild-type and *Mater*^*Null*^ females were fixed in 1% glutaraldehyde in PBS buffer (pH 7.4) and incubated at 4 °C for 2 h. After extensive washing in PBS buffer, the eggs were embedded in 2% agarose. The samples were then dehydrated through a graded series of ethanol solutions and processed for embedding in LR-White resin. Ultrathin sections were obtained with an ultramicrotome (Microm International GmbH) and mounted on formvar coated nickel grids. For lectin-cytochemistry, grids were incubated with WGA-HRP as previously described^17^. Ultrathin sections were counterstained with uranyl acetate followed by lead citrate and imaged in a Phillips Tecnai 12 transmission electron microscope. The percentage of WGA-positive CGs was calculated over twelve ultrathin sections of five eggs from wild-type and *Mater*^*Null*^ females. Image analysis was performed using Software Mip4 Advanced (Microm Image Processing Software, Digital Image Systems).

### Immunoblot

Eggs were lysed in 4x Tris-glycine SDS loading buffer with DTT, separated on 8, 10, and 4–12% Tris-glycine gels (Invitrogen) and electrophoretically transferred to polyvinylidene difluoride (PVDF) membranes (Invitrogen). The membranes were incubated (20 min, RT) in blocking reagent Blocking One (Nacalai Tesque), washed with Tris-buffered saline containing 0.1% Tween 20 (TBST) and incubated (4 °C, overnight) with primary antibodies against non-muscle myosin IIA (1:1000), myosin V (1:1000, kindly provided by John Hammer III, NHLBI, NIH) and α-tubulin (1:1000, Santa Cruz Biotechnology, Cat # sc-8035). The membranes were then washed in TBST and incubated (1 h, RT) with goat anti-rabbit or mouse IgG-HRP secondary antibodies, washed with TBST and developed using Amersham ECL Western Blotting Detection Reagent (GE Healthcare). Uncropped images of immunoblots are shown in Supplementary Fig. 8.

### In vitro cleavage assay

The post-fertilization cleavage of ZP2 (120 kD) results in two fragments (30 and 90 kD) rendering the zona matrix unreceptive to sperm binding. Ovulated and parthenogenetically activated eggs as well as two-cell embryos with intact zonae pellucidae from wildtype, *Mater*^*Null*^, and *Floped*^*Null*^ mice were resolved by SDS-PAGE and cleavage was analyzed by immunoblotting with monoclonal antibody m2c.2 to the C-terminus of mouse ZP2^55^. After stripping membranes with Restore Western Blot Stripping Buffer (Thermo Fisher Scientific), the membranes were reprobed with α-MATER (1:1000), α-FLOPED (1:1000) or α-tubulin (1:1000) antibody. Uncropped images of immunoblots are shown in Supplementary Fig. 8.

### Image analysis

Line profiles were generated in Fiji^56^ and measurements were exported to Excel for visualization. Kymographs analyzing the fluorescent signal over time were prepared using the Multi Kymograph tool in Fiji. To detect and measure cortical actin clearances, deconvolved images were imported to Fiji and normalized using the percentile threshold with “dark background” unchecked. Clearances were detected using the analyze particle function with a clearance area of 1–40 μm^2^ with the assumption of uniform circularity set at 0.2–1.00. Clearance measurements (number, area) were exported to Excel for statistical analysis and the frequency of clearances was determined per μm^2^ per given cell.

To follow CGs coming from TIRFM, iSIM and instant TIRF-SIM datasets, we performed semi-automated tracking using the plugin TrackMate Fiji Plugin^57^ (http://fiji.sc/TrackMate). For particle detection, the difference of gaussian (DoG) detector was used with estimated blob diameter of 0.6 μm, and an initial quality threshold of 120. The particles were further filtered using auto thresholds and linked with a simple linear assignment problem (LAP) linker. Linking and gap-closing distances were set to 1.0 μm and the maximum frame gap was set to 2.

We developed a Python library for working with TrackMate output data and performing analysis. Briefly, TrackMate data were imported and tracks assigned unique identifiers. For each track, derived values were calculated for each timepoint such as displacement and percent of max intensity. Aggregate values were then calculated per track, such as *R*^2^, slope, and p-value of linear regression of percent max intensity; bounding ellipses; duration; total displacement. Thresholds for duration classes (short, medium, long) were empirically determined based on histograms of duration. To identify tracks as approaching or vanishing, we required the linear regression of percent-of-max-intensity to have *R*^2^ > 0.6 (empirically determined based on histograms), *p* < 0.05, and either a positive (for approaching) or negative (for vanishing) slope. This algorithm can be generalized for analyses in other systems.

### Statistical analysis

Unpaired two-tailed Student’s *t* test was used for comparison of two samples with normal distribution. N-1 chi-squared test was used for comparison of percentages. *p* < 0.05 was considered significant. Statistical analyses and graphing were performed using Excel and SigmaPlot 12.3 (Systat Software). All bar and boxplot graphs presented in this study indicate the mean value and the standard error of the mean (s.e.m.).

### Reporting summary

Further information on research design is available in the Nature Research Reporting Summary linked to this article.

## Supplementary information

Supplementary Information

Description of Additional Supplementary Files

Supplementary Movie 1

Supplementary Movie 2

Supplementary Movie 3

Supplementary Movie 4

Supplementary Movie 5

Supplementary Movie 6

Supplementary Movie 7

Supplementary Movie 8

Reporting Summary

Source Data

## Data Availability

The source underlying Figs. 1d, 1i, 2d, 2f, 2h, 4c, 5c, 5f, 6b, 6d, 6e, 6h, and Supplementary Figs. 2h, 6 and 7 are provided as a Source Data File. Other data that support the findings of this study are available from the corresponding author on request.

## Code availability

A zip file of the Python library, the Jupyter notebook, the raw data and a full environment specification of the installed packages used for analysis of TrackMate output data can be found at 10.5281/zenodo.2605576.
