# Author Correction: Anchoring cortical granules in the cortex ensures trafficking to the plasma membrane for post-fertilization exocytosis

**DOI:** 10.1038/s41467-019-10935-1

**Published:** 2019-06-27

**Authors:** Edgar-John Vogt, Keizo Tokuhiro, Min Guo, Ryan Dale, Guanghui Yang, Seung-Wook Shin, Maria Jimenez Movilla, Hari Shroff, Jurrien Dean

**Affiliations:** 1 Laboratory of Cellular and Developmental Biology, NIDDK, National Institutes of Health, Bethesda, MD 20892 USA; 2 Section on High Resolution Optical Imaging, NIBIB, National Institutes of Health, Bethesda, MD 20892 USA; 3 Department of Cell Biology and Histology, Medical School, University of Murcia, IMIB, 30100 Murcia, Spain; 4 Advanced Imaging and Microscopy Resource, National Institutes of Health, Bethesda, MD 20892 USA; 5 Present Address: Charité - Universitätsmedizin Berlin, Augustenburger Platz 1, 13353 Berlin, Germany; 6 Present Address: Department of Genome Editing, Institute of Biomedical Science, Kansai Medical University, 2-5-1 Shinmachi, Hirakata, Osaka 573-1010 Japan; 7 Present Address: Bioinformatics and Scientific Programming Core, Eunice Kennedy Shriver National Institute of Child Health and Human Development, National Institutes of Health, Bethesda, MD 20892 USA

**Keywords:** Super-resolution microscopy, Actin, Exocytosis

Correction to: *Nature Communications* 10.1038/s41467-019-10171-7, published online 22 May 2019.

The original version of this Article omitted from the author list, the sixth author Seung-Wook Shin, who is from the ‘Laboratory of Cellular and Developmental Biology, NIDDK, National Institutes of Health, Bethesda, MD 20892, USA.’ In addition, the following was added to the Author contributions: ‘S.W.S. performed an immunoblot.’ This has been corrected in both the PDF and HTML versions of the Article.

