# Supplementary material for: Anchoring cortical granules in the cortex ensures trafficking to the plasma membrane for post-fertilization exocytosis
Source: Nat Commun. 2019 May 22;10:2271. doi: 10.1038/s41467-019-10171-7 (PMC6531442; doi:10.1038/s41467-019-10171-7)
Supplement: Supplementary file 1 — Supplementary Information [file 41467_2019_10171_MOESM1_ESM.docx]

**Supplementary Information**

Anchoring cortical granules in the cortex ensures trafficking to the plasma membrane for post-fertilization exocytosis

Edgar-John Vogt, Keizo Tokuhiro, Min Guo, Ryan Dale, Guanghui Yang, Maria Jimenez Movilla, Hari Shroff and Jurrien Dean

**
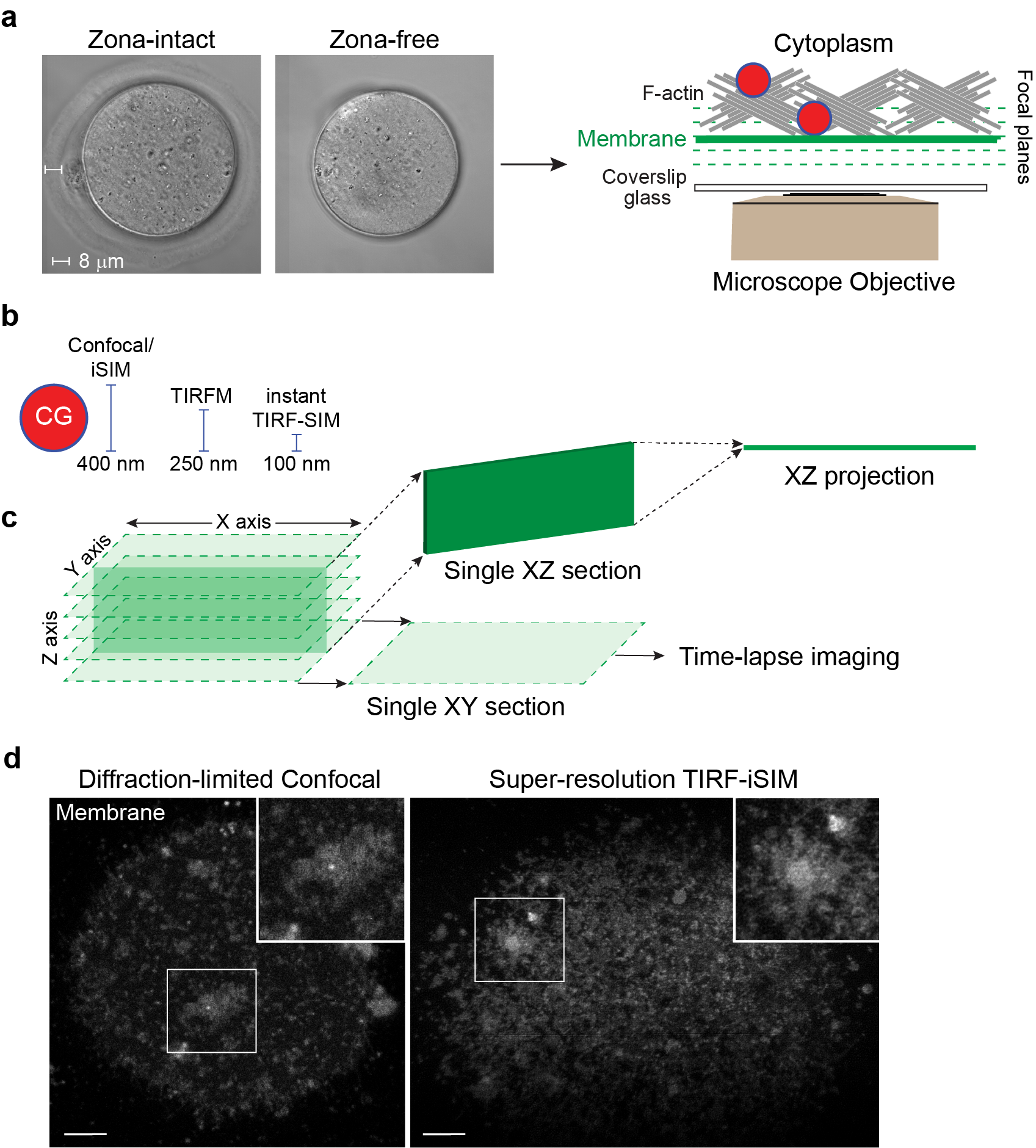
**

**Supplementary Figure 1** Diffraction-limited and super-resolution microscopy of zona-free mouse eggs. **a** Schematic representation of live-imaging zona-free mouse eggs close to the coverslip surface for diffraction-limited and super-resolution microscopy. The zona pellucida (~8 μm thick) was removed by acid Tyrode’s media and zona-free eggs were placed on poly-L-lysine coated coverslips. Transgenic mouse eggs expressed ovastacin^mCherry^ as marker of cortical granules. Eggs were stimulated with SrCl_2_ to trigger exocytosis. **b** Axial size scales when imaging mouse cortical granules (left) using high resolution confocal microscopy or instant structured illumination microscopy (iSIM), total internal reflected fluorescence microscopy (TIRFM), and super-resolution instant TIRF-SIM. CG, cortical granule. **c** Z-stacks of XY optical sections were acquired at 0.1-0.2 μm steps, from which single XZ planes (middle) were extracted and collapsed into a XZ projection (right). **d** Comparative images of zona-free mouse eggs labeled with membrane dye taken and imaged with diffraction-limited confocal microscopy and super-resolution instant TIRF-SIM. Insets correspond to higher magnification views of white rectangular regions. Scale bar, 5 μm.

**
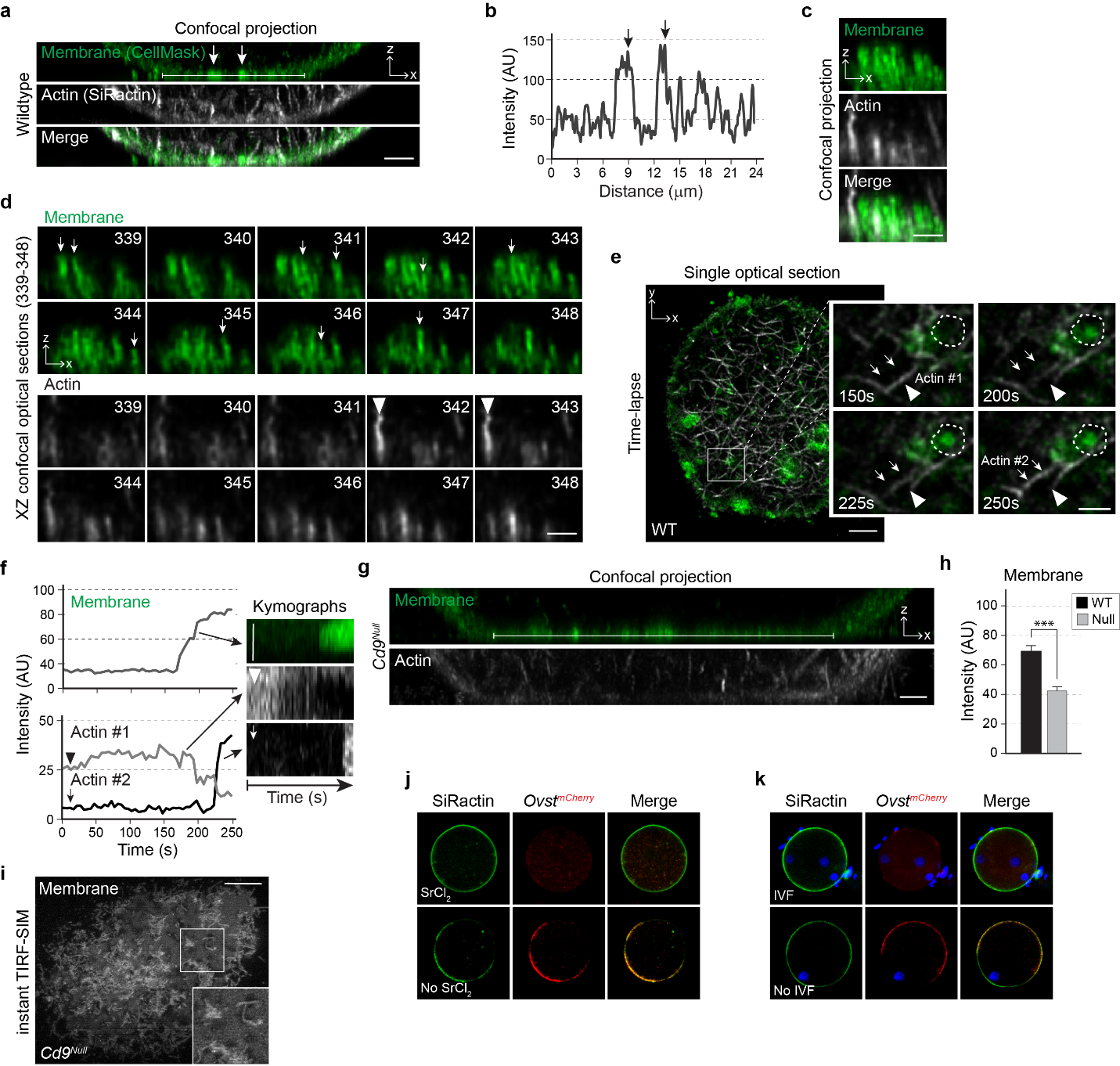
**

**Supplementary Figure 2** A dynamic actin cytoskeleton underlies the egg plasma membrane.

**a** A representative confocal image of a XZ projection of a live wildtype mouse egg. The microvillar plasma membrane was labeled with CellMask (green) and the actin cytoskeleton with the fluorogenic probe SiRactin (grey). Scale bar, 5 μm. Arrows, regions of high membrane fluorescent intensity. **b** Line profile plot of the membrane (solid line in **a**) with fluorescence intensity in arbitrary units. The two arrows highlight peaks of fluorescence corresponding to arrows in **a**. **c** Magnified XZ projection view of a cluster region. Scale bar, 2 μm. **d** Individual resliced XZ optical sections (339-348 spaced 0.1 μm apart) showing individual microvilli-like structures in the membrane (upper panels, arrows) and actin fibers (lower panels, arrowheads). Scale bar, 2 μm. **e** Time-lapse confocal images (single XY optical section) of a stimulated wildtype egg. Enlarged images (right) at indicated time points document highly dynamic cortical actin which is retracting (arrowhead) and reappearing (arrow). Plasma membrane (green) accumulates in clusters (enclosed by dashed line). Scale bars, 5 μm (left), 2 μm (right). **f** Kymographs of membrane (dashed line in **e**) and actin (arrow and arrowhead in **e**). Quantification of membrane and actin fluorescence in arbitrary units over time. Scale bar, 1 μm. **g** A representative confocal XZ projection of a *Cd9^Null^* egg. The microvillar plasma membrane was labeled with CellMask (green) and the actin cytoskeleton with the fluorogenic probe SiRactin (grey). Scale bar, 5 μm. **h** Quantification in arbitrary units of fluorescent intensity of membrane dye in wildtype (solid line in G: n=) and *Cd9^Null^* (n=13) eggs from 2 biologically independent samples. ***Ρ <0.001 by two-tailed Student’s t-test. Error bar=s.e.m. Source data are provided as a Source Data file. **i** Zona-free *Cd9^Null^* egg imaged in the presence of the membrane dye CellMask using instant TIRF-SIM. Inset corresponds to higher magnification view of white rectangular region. Scale bar, 5 μm. **j** *Ovastacn^mCherry^* (*Ovst^mCherry^*) MII eggs were activated (50 min) with 5 mM SrCl_2_ to induce cortical granule exocytosis in the presence of SiRactin (5 μm) and imaged by confocal microscopy. **k** Same as **j**, but 12 hr after *in vitro* fertilization (IVF) with capacitated mouse sperm.

**
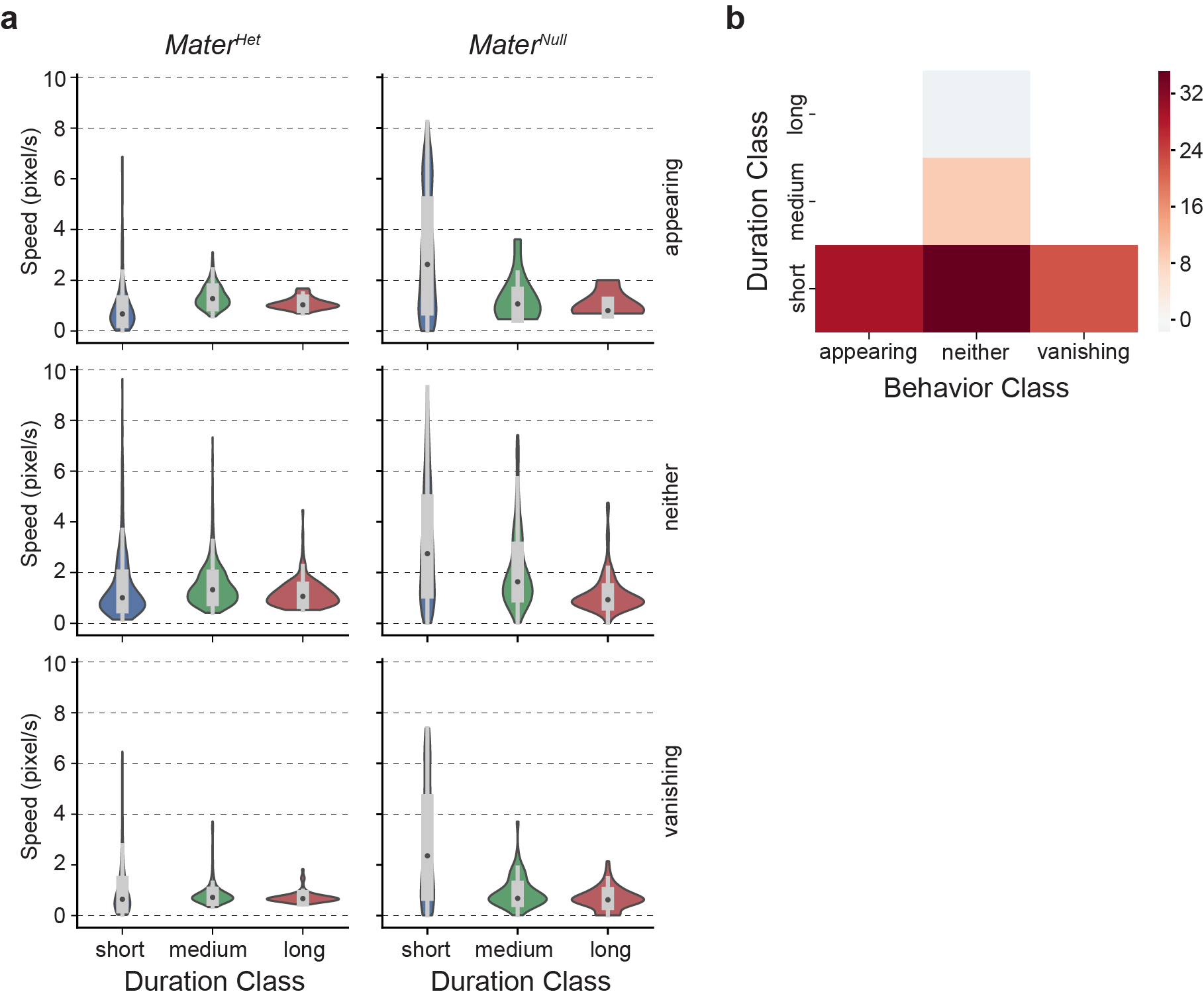
**

**Supplementary Figure 3** Short-duration cortical granules move faster at the plasma membrane in the absence of MATER. **a** Violin plots show speed (pixel/s) of short-, medium-, and long-duration tracks averaged over the length of each track in stimulated *Mater^Het^* (left) and *Mater^Null^* (right) eggs. The plot boundary captures all data points with the width reflecting the relative kernel density. Within the plot center, the thin and thick grey lines indicate 95% confidence and interquartile range, respectively. **b** Heatmap showing differences between *Mater^Het^* and *Mater^Null^* average track speeds. Rows correspond to duration classes and columns correspond to behavior classes. Each box represents a Mann-Whitney U test between the average speeds of all *Mater^Het^* and *Mater^Null^* tracks for that row and column class. Color represents -log_10_(FDR) such that darker red indicates a more significant difference in speeds.


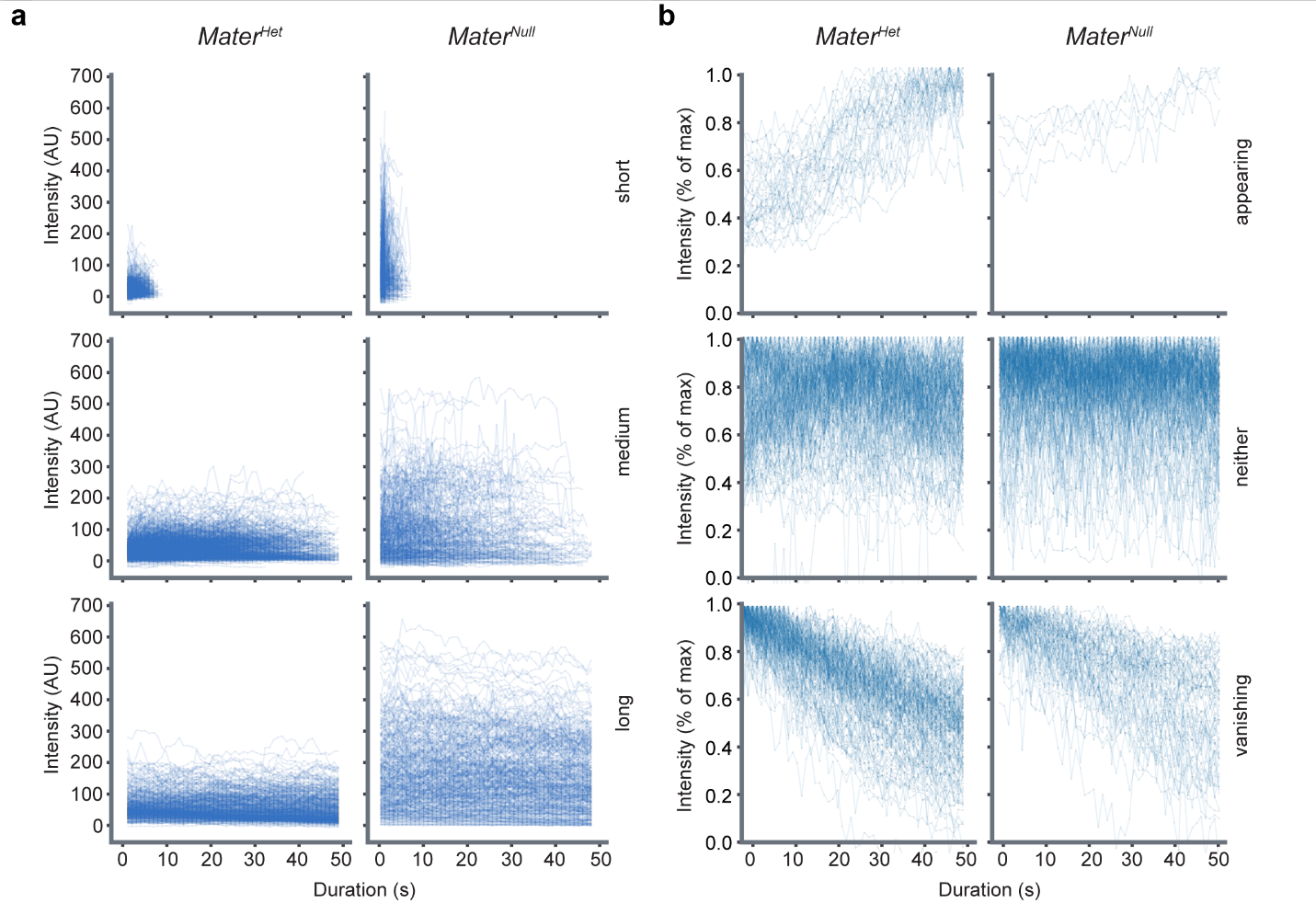


**Supplementary Figure 4** Total trafficking events of cortical granules to the plasma membrane.

**a** Graph plots show mean fluorescent intensities (arbitrary units) of total short-, medium-, and long-duration tracks in stimulated *Mater^Het^* and *Mater^Null^* eggs. **b** Graph plots show the mean intensity as a percentage of the maximum intensity of total tracks (from long-duration tracks), which are classified as appearing, neither, or vanishing in stimulated *Mater^Het^* and *Mater^Null^* eggs.

**
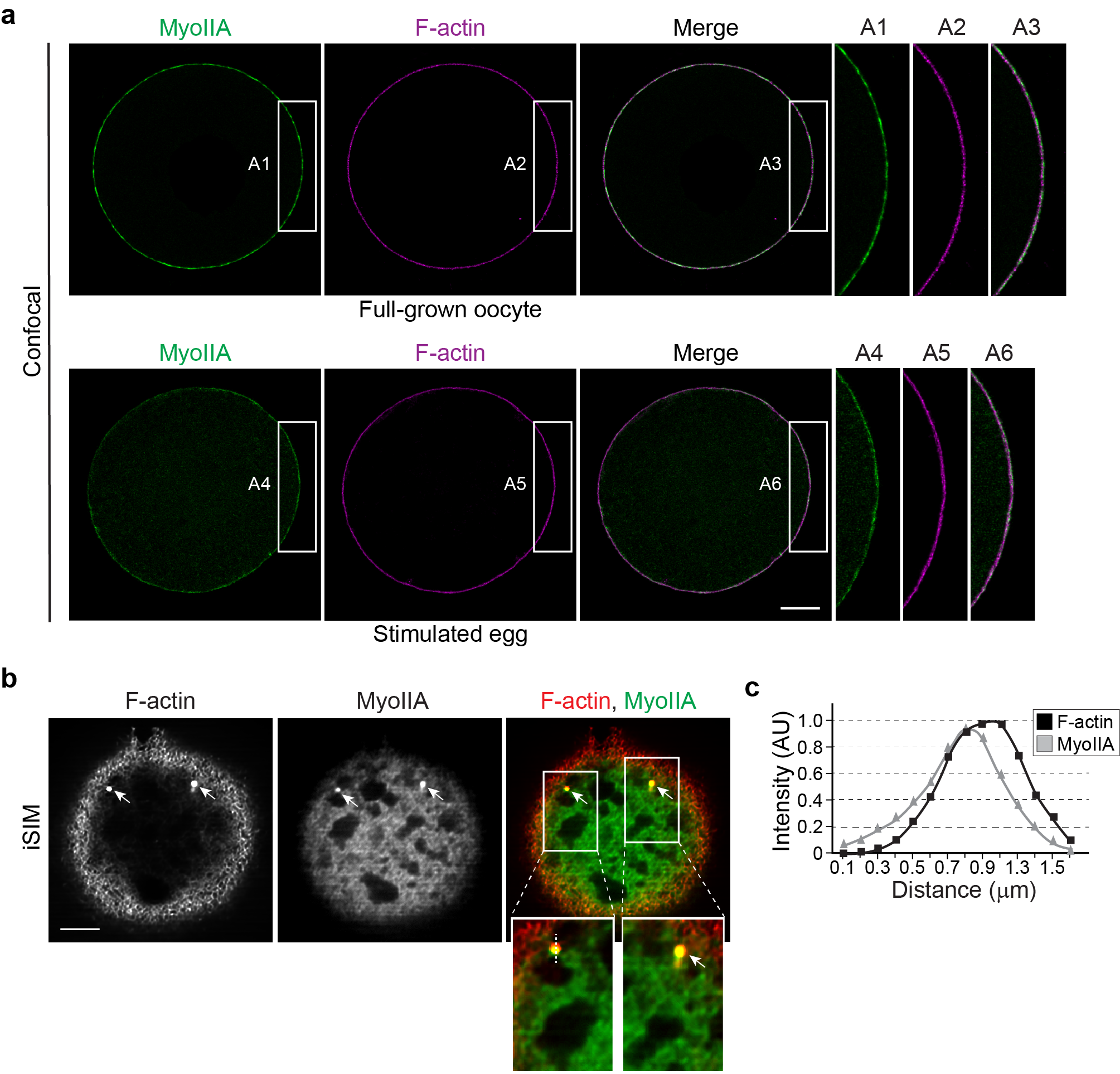
**

**Supplementary Figure 5** Myosin IIA localizes to the actin cortex in mouse eggs. **a** Full-grown oocytes (upper) and stimulated eggs (lower) were collected from transgenic females expressing myoIIA^EGFP^ (green) and fixed. F-actin in the cortex was visualized with phalloidin (magenta). Representative confocal z-projections are shown with a cropped view of the cortex (A1-A6) corresponding to white insets for the respective channels. Scale bar, 20 μm. **b** Stimulated zona-free eggs expressing myoIIA^EGFP^ were fixed and F-actin was stained with phalloidin. XY optical sections (spaced 0.2 μm apart) of the cortex were obtained with super-resolution iSIM. Representative iSIM projection of the cortical region shows F-actin, myoIIA^EGFP^ and a merged image with enlarged views of two regions of interest (below). Arrows, F-actin and EGFP puncta. Scale bar, 5 μm. **c** Profile plot of dashed line in **b** indicating peaks in F-actin and EGFP channel.

**
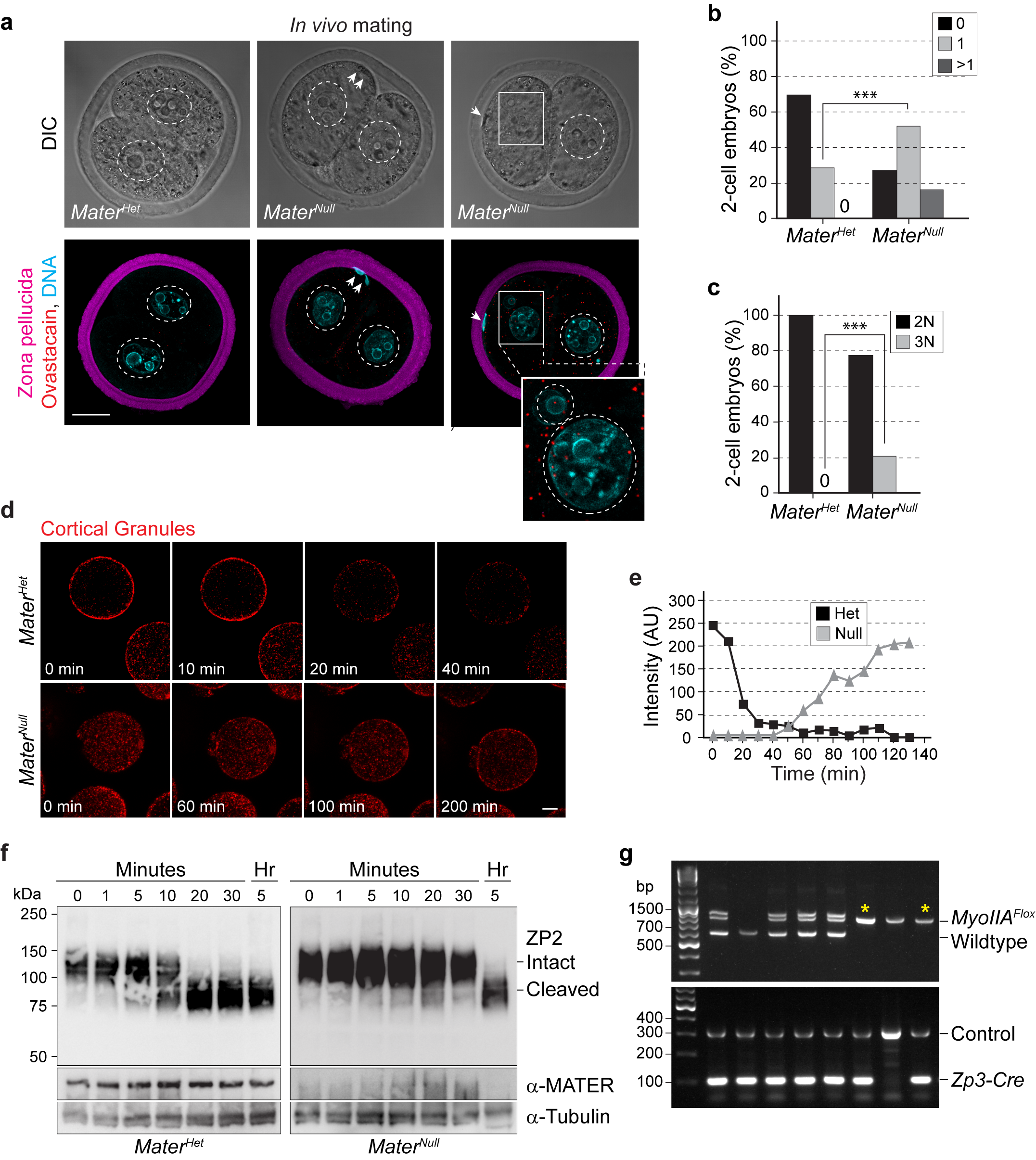
**

**Supplementary Figure 6** MATER and myosin IIA are required to prevent polyspermy in early embryos. **a** Hormonally stimulated *Mater^Het^* and *Mater^Null^* female mice expressing ovastacin^mCherry^ (red) were mated with wildtype males to collect 2-cell embryos which were fixed. In representative confocal cross-sections of z-projections, zonae pellucidae were visualized with conjugated WGA-Alexa Fluor 633 (magenta) and DNA was stained with DAPI (cyan). Arrows, supernumerary sperm in the perivitelline space (PVS); dashed lines, nuclei. Polyspermic embryo in inset. Scale bar, 20 μm. **b** The percent of embryos with 0, 1 or 2 sperm in the PVS was determined for each genotype using 7-11 embryos from 2 biologically independent samples. ***Ρ <0.001 by Chi-squared test. **c** Same as **b**, but for the number of nuclei. ***Ρ <0.001 by Chi-squared test. **d** Live confocal imaging of ovulated eggs from *Mater^Het^* (upper) and *Mater^Null^* (lower) female mice expressing ovastacin^mCherry^ after stimulation with SrCl_2_ to trigger exocytosis (see corresponding Supplementary Videos 7,8). Representative time-lapse optical sections at indicated time points. Scale bar, 20 μm. **e** Mean fluorescent intensities (arbitrary units) of ovastacin^mCherry^ in the cortical region of *Mater^Het^* (black, square) and *Mater^Null^* (grey, arrowhead) eggs from **d** were measured and plotted as a function of time to document exocytosis. **f** Immunoblot of lysates from stimulated eggs (10) collected from *Mater^Het^* (left) and *Mater^Null^* (right) female mice at indicated time points and probed with mAb specific to C-terminal region of mouse ZP2. Intact ZP2, 120 kDa; cleaved ZP2, 90 kDa. Immunoblots were probed with antibodies to MATER and α-tubulin to confirm the genotype and serve as load controls, respectively. **g** PCR genotyping of offspring’s tails from mating *MyoIIA^Flox/Flox^* female mice with *MyoIIA^Flox/Flox^/Zp3^Cre/+^* male mice to obtain *MyoIIA^Flox/Flox^/Zp3^Cre/+^* female (yellow asterisk). *MyoIIA* floxed allele (770 bp), wildtype allele (600 bp), and *Cre* allele (100 bp) were detected with corresponding primers (Supplementary Table 1). Source data are provided as a Source Data file.

**
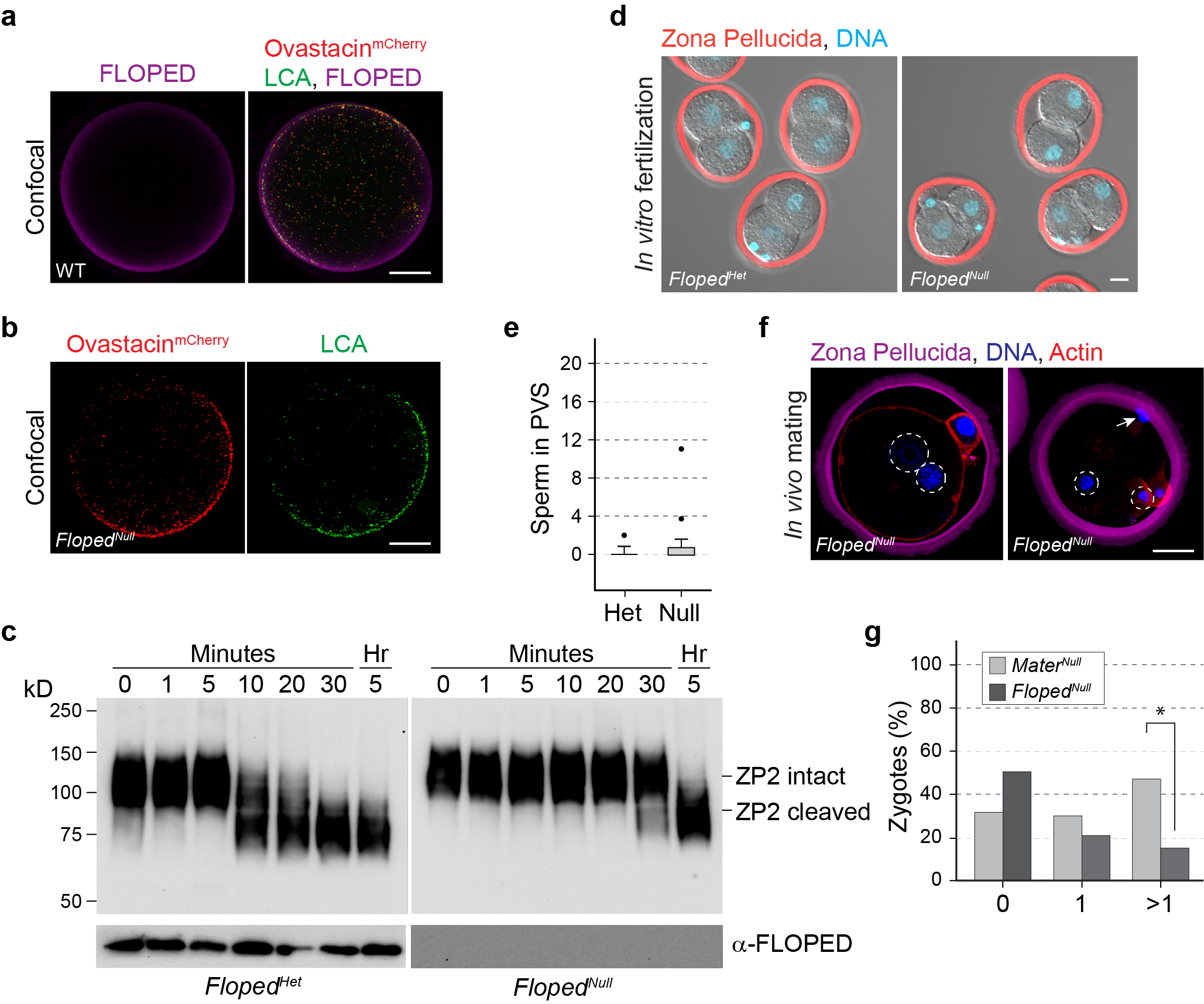
**

**Supplementary Figure 7** FLOPED is not required for the zona pellucida block to polyspermy.

**a** Ovulated eggs were collected from wildtype females expressing ovastacin^mCherry^ (red), fixed and immunostained with anti-FLOPED (magenta). Cortical granules were stained with conjugated lectin LCA-FITC (green) and imaged by confocal microscopy. Representative confocal cross-sections of z-projections including a merged image are shown. Scale bar, 20 μm. **b** Same as **a**, but for *Floped^Null^* eggs. **c** Immunoblot of lysates from stimulated eggs (30) collected from *Floped^Het^* (left) and *Floped^Null^* (right) female mice at indicated time points and probed with mAb specific to the C-terminal region of mouse ZP2. Intact ZP2, 120 kDa; cleaved ZP2, 90 kDa. Immunoblots were probed with antibodies to FLOPED and α-tubulin to confirm the genotype and serve as load controls, respectively. **d** Ovulated eggs from *Floped^Het^* (left) and *Floped^Null^* (right) female mice were inseminated and cultured to 2-cell embryos. Zonae pellucidae were visualized with the lectin WGA- Alexa Fluor 633 (red) and sperm DNA was stained with Hoechst 33342 (blue). Scale bar, 20 μm. **e** The number of sperm observed in the PVS after *in vitro* fertilization for each genotype using 20-30 2-cell embryos from 3 biologically independent samples. Box plot includes the mean (horizontal line) and data between the 25^th^ and 75^th^ percentile. Error bars indicate the 90^th^ and 10^th^ percentiles and outliers are indicated by dots. **f** Hormonally stimulated *Floped^Null^* female mice were mated with wildtype males. Fertilized zygotes were collected and fixed. Zonae pellucidae were visualized with WGA-Alexa Fluor 633 (magenta), F-actin in the cortex was stained with phalloidin (red) and DNA was stained with DAPI (blue). Representative confocal cross sections of z-projections are shown. Arrows, supernumerary sperm in the PVS; dashed line, pronuclei. Scale bar, 20 μm. **g** The number of sperm observed in the PVS was determined for *Floped^Null^* in **f** using 15 embryos from 2 biologically independent samples and compared to *Mater^Null^* (Fig. 6c, d). 0.62 ± 0.21 (*Floped^Null^*) vs. 1.82 ± 0.25 (*Mater^Null^*). *Ρ <0.05 by Chi-squared test. Source data are provided as a Source Data file.

**
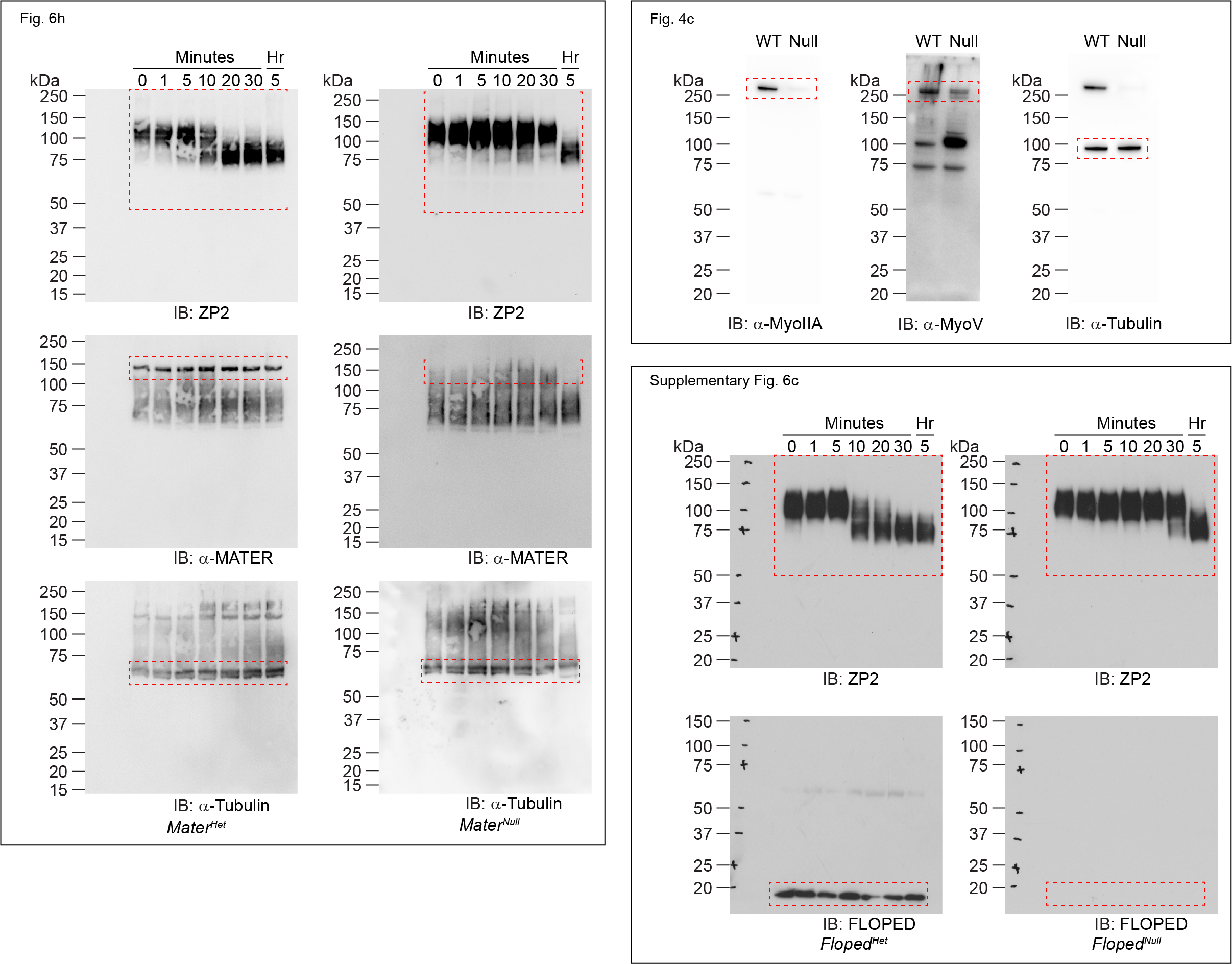
**

**Supplementary Figure 8** Uncropped images of immunoblots. Uncropped images of immunoblots displayed in figures. Red boxes indicate areas that were cropped in corresponding figure.

**Supplementary Table 1. Primers for genotyping transgenic mice**

| **Gene** | **Sequence (5’ to 3’)** |  | **Amplicon size (bp)** |
| --- | --- | --- | --- |
| *Ovastacin^mCherry^* | AAGCCAGCACCTATCCAAGA | F^1^ | 976 (with mCherry)  268 (without mCherry) |
|  | GGGTGTTGTGGGAAAAGAGA | R |  |
| *Mater* | TCATGTCCTTGGATGGCATG | F | 242 (Null)  390 (WT^2^) |
|  | ACCGGTGGATGTGGAATGTG | F |  |
|  | CCACGTGCTTTCAAGATTGC | R-common |  |
| *Floped* | CCCTGCTGACAGTGGACTC | F | 890 (Null)  512 (WT) |
|  | CCAGCCAGTTTTAGCCCTTT | F |  |
|  | TGCGCAACTGTTGGGAAG | R-common |  |
| *MyosinIIA^EGFP^* | CTGTCACATGGCTCATGTTC | F | 200 (Knockin)  400 (WT) |
|  | GCCGGACACGCTGAACTTGT | F |  |
|  | GCCCTGAGTAGTATCGCTCC | R-common |  |
| *MyosinIIA^Flox^* | GGGACACAGTTGAATCCCTT | F | 770 (Floxed)  600 (WT) |
|  | ATGGGCAGGTTCTTATAAGG | R |  |
| *Cd9^Null^* | CTGGTCACACCCCCTAACGGAGC | F | 500 (Null) |
|  | GCTTGGCGGCGAATGGGCTGA | R |  |
| *Cd9^WT^* | TGCAGGCATGGAGGCGCAGC | F | 375 (WT) |
|  | GTGCCGGCCTCGCCTTTCCC | R |  |
| *Zp3^Cre^* | GCGGTCTGGCAGTAAAAACTATC | F | 100 (TG) |
|  | GTGAAACAGCATTGCTGTCACTT | R |  |
|  | GTAGGTGGAAATTCTAGCATCATCC | R |  |

^1^F, forward; R, reverse

^2^WT, wildtype**Supplementary Table 2. Parameters of imaging experiments**

| **Figure** | **Method** | **Frame Rate** | **Duration** | **Pixel Size** |
| --- | --- | --- | --- | --- |
| 1**a** | Confocal | Fixed | - | 94 nm xy, 600 nm z |
| 1**b** | Confocal | Live | - | 60 nm xy, 200 nm z |
| 1**c** | Confocal | Every 5 s | 250 s | 60 nm xy |
| 1**e** | iSIM | Fixed | - | 64 nm xy, 100 nm z |
| 1**g** | iSIM | Every 10 s | 5 min | 107 nm xy, 500 nm z |
| 2**a**, 2**b** | Confocal | Fixed | - | 94 nm xy, 600 nm z |
| 2**c** | Confocal | Live | - | 60 nm xy, 200 nm z |
| 2**e** | iSIM | Fixed | - | 64 nm, 100 nm z |
| 3**a** | instant TIRF-SIM | Every 1 s | 50 s | 33.4 nm xy |
| 4**a** | TIRFM | Every 10 s | 300 s | 254 nm xy |
| 4**d** | Confocal | Fixed | - | 101 nm xy, 505 nm z |
| 4**e** | iSIM | Fixed | - | 107 nm xy, 200 nm z |
| 5 | iSIM | Fixed | - | 64 nm xy, 100 nm z |
| 6**a** | Confocal | Fixed | - | 0.593 μm xy |
| 6**c**, 6**f**, 6**g** | Confocal | Fixed | - | 86.5 nm xy, 1 μm z |
| S1**d** | instant TIRF-SIM | Frame | - | 33.4 nm xy |
| S2**a** | Confocal | Live | - | 60 nm xy, 200 nm z |
| S2**e** | Confocal | Every 5 s | 250 s | 60 nm xy |
| S2**g** | Confocal | Live |  | 60 nm xy, 200 nm z |
| S2**i** | instant TIRF-SIM | Frame | - | 33.4 nm xy |
| S5**a** | Confocal | Fixed | - | 104 nm, 1μm z |
| S5**b** | iSIM | Fixed | - | 107 nm xy, 200 nm z |
| S6**a** | Confocal | Fixed | - | 86.5 nm xy, 1 μm z |
| S6**d** | Confocal | Every 10 min | 200 min | 0.593 μm xy, 2 μm z |
| S7**a**, S7**b** | Confocal | Fixed | - | 94 nm xy, 600 nm z |
| S7**d** | Confocal | Fixed | - | 0.593 μm xy |
| S7**f** | Confocal | Fixed | - | 86.5 nm xy, 1 μm z |
