# Supplementary material for: Anchoring cortical granules in the cortex ensures trafficking to the plasma membrane for post-fertilization exocytosis
Source: Nat Commun. 2019 May 22;10:2271. doi: 10.1038/s41467-019-10171-7 (PMC6531442; doi:10.1038/s41467-019-10171-7)
Supplement: Supplementary file 2 — Description of Additional Supplementary Files [file 41467_2019_10171_MOESM2_ESM.docx]

**Description of Additional Supplementary Files**

File Name: Supplementary Movie 1

Description: Single cortical granule captured and pulled by actin. Live confocal imaging of zona-free eggs expressing ovastacin^mCherry^ (red) in a single optical section after stimulation with SrCl_2_ to trigger exocytosis in the presence of the fluorogenic probe SiRactin (grey) for F-actin and the membrane dye CellMask (green).

File Name: Supplementary Movie 2

Description: Single mouse cortical granule moving along actin. Live confocal imaging of zona-free eggs expressing ovastacin^mCherry^ (red) in a single optical section after stimulation with SrCl_2_ to trigger exocytosis in the presence of the fluorogenic probe SiRactin (grey) for F-actin and the membrane dye CellMask (green).

File Name: Supplementary Movie 3

Description: Mouse cortical granules accumulate in the egg cortex. Live iSIM imaging of zona-free eggs expressing ovastacin^mCherry^ (red) after stimulation with SrCl_2_ to trigger exocytosis in the presence of the membrane dye CellMask (green). Movie depicts an XZ projection.

File Name: Supplementary Movie 4

Description: Trafficking of mouse cortical granules at the plasma membrane in the presence of MATER. Live instant TIRF-SIM imaging of zona-free eggs from *Mater^Het^* female mice expressing ovastacin^mCherry^ (red) after stimulation with SrCl_2_ to trigger exocytosis in the presence of the membrane dye CellMask (green). Merged Movie is shown on the left; ovastacin^mCherry^ channel in grayscale is on the right.

File Name: Supplementary Movie 5

Description: Trafficking of mouse cortical granules at the plasma membrane in the absence of MATER. Live instant TIRF-SIM imaging of zona-free eggs from *Mater^Null^* female mice expressing ovastacin^mCherry^ (red) after stimulation with SrCl_2_ to trigger exocytosis in the presence of the membrane dye CellMask (green). Merged Movie is shown on the left; ovastacin^mCherry^ channel in grayscale is on the right.

File Name: Supplementary Movie 6

Description: Non-muscle myosin IIA associates with mouse cortical granules. Live TIRFM imaging of zona-free eggs expressing ovastacin^mCherry^ (red) and myoIIA^EGFP^ after stimulation with SrCl_2_ to trigger exocytosis in the presence of the fluorogenic probe SiRactin (magenta) for F-actin.

File Name: Supplementary Movie 7

Description: Cortical granule exocytosis in the presence of MATER. Live confocal imaging of ovulated eggs from *Mater^Het^* female mice expressing ovastacin^mCherry^ (red) after stimulation with SrCl_2_ to trigger exocytosis

File Name: Supplementary Movie 8

Description: Cortical granule exocytosis in the absence of MATER. Live confocal imaging of ovulated eggs from *Mater^Null^* female mice expressing ovastacin^mCherry^ (red) after stimulation with SrCl_2_ to trigger exocytosis.
